# Perceived need and barriers to adolescent mental health care: agreement between adolescents and their parents

**DOI:** 10.1017/S2045796019000568

**Published:** 2019-09-20

**Authors:** N. Schnyder, D. Lawrence, R. Panczak, M. G. Sawyer, H. A. Whiteford, P. M. Burgess, M. G. Harris

**Affiliations:** 1 School of Public Health, The University of Queensland, Brisbane, Australia; 2 Policy and Epidemiology Group, Queensland Centre for Mental Health Research, Brisbane, Australia; 3 Graduate School of Education, The University of Western Australia, Perth, Australia; 4 Queensland Centre for Population Research, School of Earth and Environmental Science, The University of Queensland, Brisbane, Australia; 5 School of Medicine, University of Adelaide, Adelaide, SA, Australia; 6 Research and Evaluation Unit, Women's and Children's Health Network, Adelaide, SA, Australia; 7 Institute for Health Metrics and Evaluation, University of Washington, Seattle, Washington, USA

**Keywords:** Adolescent mental health care, agreement adolescents and parents, barriers, perceived need

## Abstract

**Aims:**

Mental disorders cause high burden in adolescents, but adolescents often underutilise potentially beneficial treatments. Perceived need for and barriers to care may influence whether adolescents utilise services and which treatments they receive. Adolescents and parents are stakeholders in adolescent mental health care, but their perceptions regarding need for and barriers to care might differ. Understanding patterns of adolescent-parent agreement might help identify gaps in adolescent mental health care.

**Methods:**

A nationally representative sample of Australian adolescents aged 13–17 and their parents (*N* = 2310), recruited between 2013–2014, were asked about perceived need for four types of adolescent mental health care (counselling, medication, information and skill training) and barriers to care. Perceived need was categorised as fully met, partially met, unmet, or no need. Cohen's kappa was used to assess adolescent-parent agreement. Multinomial logistic regressions were used to model variables associated with patterns of agreement.

**Results:**

Almost half (46.5% (s.e. = 1.21)) of either adolescents or parents reported a perceived need for any type of care. For both groups, perceived need was greatest for counselling and lowest for medication. Identified needs were fully met for a third of adolescents. Adolescent-parent agreement on perceived need was fair (kappa = 0.25 (s.e. = 0.01)), but poor regarding the extent to which needs were met (kappa = −0.10 (s.e. = 0.02)). The lack of parental knowledge about adolescents' feelings was positively associated with adolescent-parent agreement that needs were partially met or unmet and disagreement about perceived need, compared to agreement that needs were fully met (relative risk ratio (RRR) = 1.91 (95% CI = 1.19–3.04) to RRR = 4.69 (95% CI = 2.38–9.28)). Having a probable disorder was positively associated with adolescent-parent agreement that needs were partially met or unmet (RRR = 2.86 (95% CI = 1.46–5.61)), and negatively with adolescent-parent disagreement on perceived need (RRR = 0.50 (95% CI = 0.30–0.82)). Adolescents reported most frequently attitudinal barriers to care (e.g. self-reliance: 55.1% (s.e. = 2.39)); parents most frequently reported that their child refused help (38.7% (s.e. = 2.69)). Adolescent-parent agreement was poor for attitudinal (kappa = −0.03 (s.e. = 0.06)) and slight for structural barriers (kappa = 0.02 (s.e. = 0.09)).

**Conclusions:**

There are gaps in the extent to which adolescent mental health care is meeting the needs of adolescents and their parents. It seems important to align adolescents' and parents' needs at the beginning and throughout treatment and to improve communication between adolescents and their parents. Both might provide opportunities to increase the likelihood that needs will be fully met. Campaigns directed towards adolescents and parents need to address different barriers to care. For adolescents, attitudinal barriers such as stigma and mental health literacy require attention.

## Introduction

Six of the ten leading causes of disease burden in adolescents are mental and substance use disorders or related problems (Gore *et al*., 2011). Evidence-based interventions can reduce this burden (Campion *et al*., 2012) but studies suggest that adolescents often underutilise potentially beneficial treatments (Merikangas *et al*., 2011; Sawyer *et al*., 2018). Perceived need for care and barriers to care are among the many factors that may influence whether adolescents utilise services and which treatments they receive. Adolescents and parents are stakeholders in adolescent mental health care, but their perceptions regarding need for and barriers to care might differ. Understanding patterns of adolescent-parent agreement on perceived need and barriers to care might, therefore, help to identify gaps in adolescent mental health care.

Perceived need is an individual's view about whether and what type of care they believe they need (Meadows *et al*., 2000). Consideration of the individual's preferences is an important element of patient-centred care (Epstein and Street, 2011). Approaches to assessing perceived need among adolescents have varied. For example, one study used a single question asking adolescents and their parents, whether they thought the adolescent needed mental health care (Ezpeleta *et al*., 2002). Another asked parents whether they perceived a need for any of four types of help for their child and, if they received that help, whether needs were fully or only partially met (Johnson *et al*., 2018). This latter, more detailed approach may be useful for designing patient-centred services because it considers the individuals' preferences for treatment and their views about the services that were delivered. Moreover, despite perceiving a need for care or even seeking care, adolescents' needs may be unmet or only partially met because barriers stand in their way. Understanding barriers to care may assist in developing and targeting strategies to facilitate the use of appropriate services (Reardon *et al*., 2017; Sheppard *et al*., 2018).

Parents are key gatekeepers for adolescent mental health care. However, patient-centred care approaches recognise the importance of both adolescents' and parents' perspectives but balancing them is challenging (Gondek *et al*., 2017). As introspection is developing during adolescence, adolescents might perceive a need for care for different problems than their parents because parents have to rely on adolescents' observable behaviour (De Los Reyes *et al*., 2015) to detect a need. Adolescents might also perceive needs for different types of care than their parents depending on their knowledge about treatment options or aetiology of mental problems (Farrer *et al*., 2008). Even when treatment is received, adolescents and parents may evaluate the benefits of that care differently (Aarons *et al*., 2010). Ideally, adolescent mental health services should aim to meet the treatment needs of adolescents as perceived by both adolescents and their parents. Therefore, one possible approach to reveal gaps in adolescent care is to understand patterns of adolescent-parent agreement on perceived need for care. For example, adolescent-parent agreement that perceived needs were fully met may indicate that there was no gap in the extent to which adolescents' mental health care met needs. Agreement that perceived need was only partially met or unmet, or disagreement about the extent to which need was met, may indicate unmet need and gaps in adolescents' mental health care. To date, the few studies that have examined perceived need and barriers to adolescent mental health care from both the adolescents' and parents' perspectives (Ezpeleta *et al*., 2002; Williams *et al*., 2011) have relied on selective samples with limited implications for policy or service development.

Our understanding of who is more likely to perceive unmet need may be enhanced by exploring the factors associated with patterns of agreement. The presence or type of mental disorder has been associated with a perceived need for care (Codony *et al*., 2009; Johnson *et al*., 2018), but its association with agreement is unclear. Difficulty in communicating feelings within the family has been associated with adolescent-parent disagreement on symptoms (Bidaut-Russell *et al*., 1995; Van Roy *et al*., 2010) but its relationship with agreement on perceived need has not been explored.

Finally, as with perceived need, adolescents and their parents might identify different barriers to care, as a result of their unique expectations, experiences and cognitions. As yet, however, no studies have examined this.

Using data from a nationally representative survey, we addressed three research questions to fill outlined gaps in knowledge:

1. What is the level of agreement between adolescents and their parents on perceived need for any type and different types of adolescents' mental health care?
2. What factors are associated with patterns of adolescent-parent agreement on perceived need?
3. What is the level of agreement between adolescents and their parents on barriers to care?

## Methods

### Participants and procedure

The Second Australian Child and Adolescent Survey of Mental Health and Wellbeing (or Young Minds Matter (YMM) Survey) was conducted between June 2013 and April 2014. The survey methodology is detailed elsewhere (Hafekost *et al*., 2016). Briefly, area-based random sampling was used to identify households with at least one child aged 4–17. If more than one eligible child lived in the household, the study child was selected randomly. Trained interviewers conducted face-to-face interviews with one parent or primary carer (hereafter, parents); the adolescent completed a self-report questionnaire on a tablet computer. Of eligible households, 6310 parents participated in the YMM survey with an overall response rate of 55%. The current study focused on the subset of adolescents aged 13–17. Of 2604 adolescents approached, 2314 (89%) participated. We excluded four participants (0.17%) due to missing data, two each in two of the independent variables, resulting in a total study sample of 2310. We assumed that deleting missing cases in a sample of this size would not result in a loss of precision or power, that the data was missing completely at random and that the reasons for the missing data were unrelated to the outcome. In this case, missing data in the independent variable does not lead to biased results (Sterne *et al*., 2009). The Ethics Committee of the Australian Government Department of Health approved the survey and the Human Ethics Research Office of the University of Queensland exempted the current study from ethics review.

### Measures

#### Perceived need for care

**Fig. 1.**
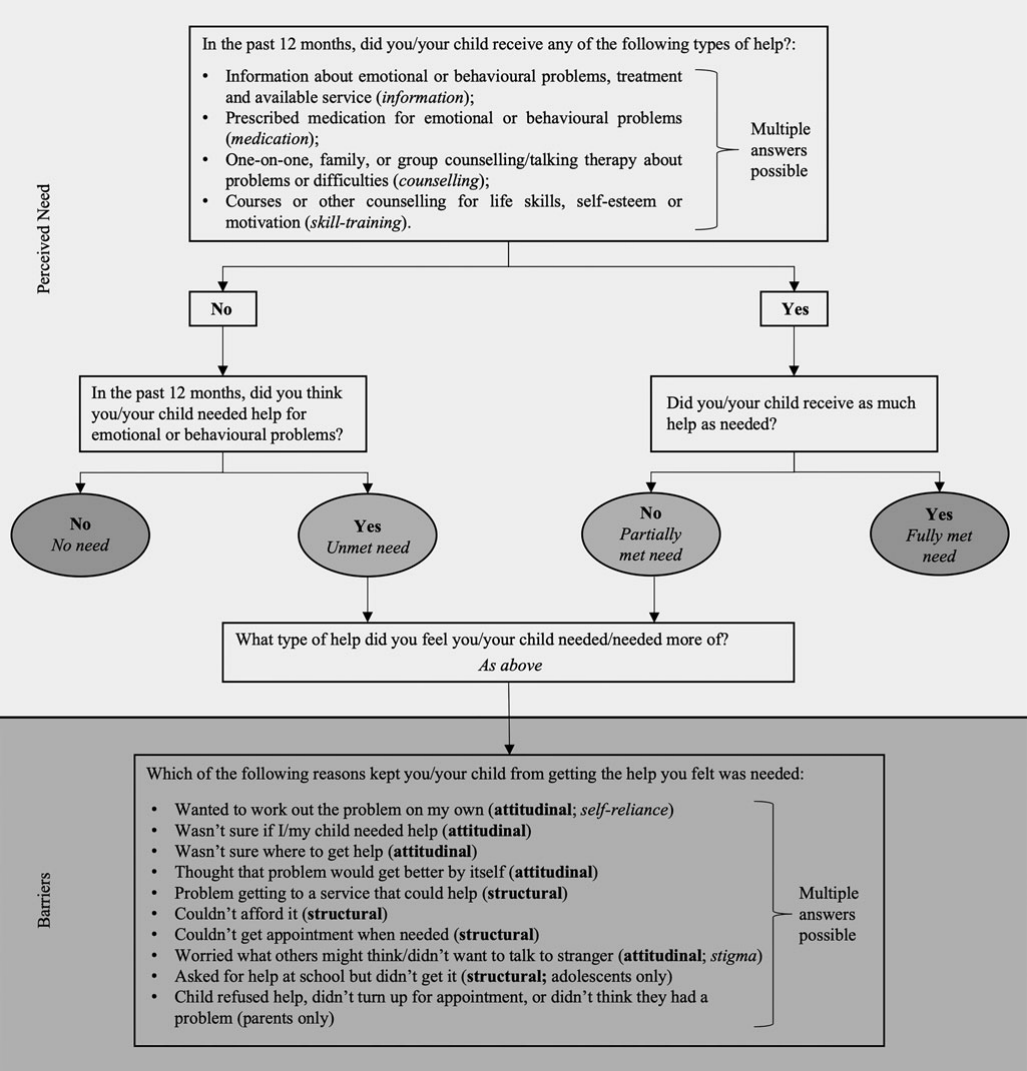
Assessment of perceived need for and barriers to care among adolescents and their parents.

Perceived need for adolescent mental health care in the past 12 months was assessed with a modified version of the Perceived Need for Care Questionnaire (Meadows *et al*., 2000) (Fig. 1). Questions were addressed to all adolescents and parents that identified an emotional or behavioural problem and additionally to parents that reported possible mental health problems in earlier questions (Johnson *et al*., 2018). First, participants were asked whether the adolescent had received help from any of four broad types of help for emotional or behavioural problems: information, medication, counselling and skill-training. For each type of help received, participants were asked whether they received enough. Second, participants who did not report having received help were asked if they thought they needed any of the same four types of help. From this information, the level of perceived need for each type of help was classified as either fully met, partially met, unmet or no need. A composite variable ‘perceived need for any type of help’ with the same levels of need was provided; it takes the greatest level of need for each type of help endorsed (Johnson *et al*., 2018).

For the current study, we categorised agreement between adolescents and their parents on the level of perceived need into a ‘patterns of agreement’ variable which had five groups: (1) agreed that needs were fully met, (2) agreed that needs were partially met or unmet, (3) adolescent perceived a greater level of need than their parent, (4) parent perceived a greater level of need than the adolescent and (5) agreed that there was no need (online Supplementary eTable 1). Groups (1)–(4) include those where either adolescents *or* parents reported a perceived need.

#### Barriers to care

Adolescents and parents who reported an unmet or a partially met need for any type of help were asked which of nine barriers kept the adolescent from getting the help they felt was needed. Most barriers were categorised as attitudinal or structural (Mojtabai *et al*., 2011) (Fig. 1). Eight of the barriers were the same for both adolescents and parents, adolescents were asked about one additional structural barrier and parents were asked whether their ‘child refused help’ which we could not conclusively categorise and therefore treat separately.

#### Additional measures

Adolescents' probable mental disorder (hereafter, probable disorder) in the past six months, parental knowledge about adolescents' feelings (an indicator for communication of feelings between adolescents and parents), family functioning, parental psychopathology, family type, remoteness, socio-economic advantage and disadvantage, parental education and adolescents' sex were additionally assessed (online Supplementary eMaterial 1). Where applicable, we provide Cronbach's *α* for scales used in this study in online Supplementary eMaterial 1.

### Statistical analyses

Survey weights were applied to the data to represent the estimated population of interest as of 30 June 2013 and analyses accounted for clustering. We computed group comparisons with the second-order Rao and Scott correction (Rao and Scott, 1984) of Pearson *χ*^2^ statistics that is converted into *F* statistics and report Cramer's *V* as a measure for the strength of association for each comparison. We computed adolescent-parent agreement on perceived need using kappa statistics (Cohen, 1960) and percentage agreement. Kappa is more robust than percentages because it accounts for agreement by chance. Kappa values of <0.00, 0.00–0.20, 0.21–0.40, 0.41–0.60, 0.61–0.80 and 0.81–1.00 were considered to represent poor, slight, fair, moderate, substantial and almost perfect agreement, respectively (Landis and Koch, 1977).

We modelled associations of adolescents' probable disorder and parental knowledge about adolescents' feelings (independent variables) with patterns of agreement on perceived need for any type of help (dependent variable). We excluded pairs that agreed on no need because they are not at risk of unmet need. We controlled for family and socio-demographic characteristics that have previously been associated with adolescent-parent disagreement on mental disorder (Grills and Ollendick, 2002; De Los Reyes and Kazdin, 2006; Chen *et al*., 2017; Bajeux *et al*., 2018), with simultaneous multinomial logistic regressions. Specified post-hoc, we ran the same model but with the adolescents' probable disorder variable replaced with separate variables measuring probable internalising and externalising disorder (see online Supplementary eMaterial 1 for categorisation of these two variables). Statistical analyses and graphs were performed in Stata version 15 (StataCorp) using the svy prefix for survey data and R version 3.5.1 (R Core Team) using the packages ‘ggplot2’ (Wickham, 2016) and ‘survey’ (Lumley, 2004).

## Results

In the total sample, a probable disorder was reported by 16.7% of adolescents or parents and little/no parental knowledge about adolescents' feelings by 31.3% of adolescents (sample characteristics in online Supplementary eTable 2).

### Level of adolescent-parent agreement on perceived need for any type and different types of adolescents' mental health care

Table 1 shows the distributions of levels of perceived need for any type of help and for each type of help, separately for adolescents and parents. Approximately one-third of both adolescents and parents reported a perceived need for any type of help. For both groups, perceived need was highest for counselling and lowest for medication. Table 1 also shows that almost half (46.5%, s.e.  =  1.21) of either adolescents *or* parents reported a perceived need for any type of help. Among this sub-sample, identified needs were fully met in around one-third of cases, varying somewhat according to the type of help needed. In the sub-sample of adolescents with a probable disorder, perceived need for any and each type of help was more frequent than in the total sample, but patterns were otherwise similar (online Supplementary eTable 3).

**Table 1.** Distribution and comparison of past 12 months perceived need among adolescents (aged 13–17) and their parents

Level of perceived need, by type of help	Total sample (*N* = 2310)		Sub-samples, either adolescent or parent identified a perceived need	
	Adolescents, % (s.e.)	Parents, % (s.e.)	Adolescents, % (s.e.)	Parents, % (s.e.)
Any type of help			*n* = 1119 (46.5% (s.e. = 1.21))^a^	
No need	67.1 (1.11)	69.0 (1.11)	29.4 (1.62)	33.3 (1.58)
Fully met need	16.9 (0.86)	14.4 (0.81)	36.3 (1.62)	31.1 (1.54)
Partially met need	9.1 (0.64)	9.0 (0.64)	19.5 (1.28)	19.2 (1.30)
Unmet need	6.9 (0.59)	7.6 (0.67)	14.8 (1.21)	16.4 (1.34)
	*F*_(8.80,4806.59)_ = 38.39; *V* = 0.24		*F*_(8.79,4818.14)_ = 32.78; *V* = 0.32	
Counselling			*n* = 821 (34.9% (s.e. = 1.12))^a^	
No need	79.1 (0.95)	74.1 (1.08)	39.9 (1.98)	25.6 (1.68)
Fully met need	10.5 (0.71)	12.2 (0.79)	30.3 (1.80)	34.9 (1.90)
Partially met need	4.4 (0.46)	6.1 (0.55)	12.6 (1.26)	17.6 (1.49)
Unmet need	6.0 (0.54)	7.6 (0.64)	17.2 (1.47)	21.9 (1.66)
	*F*_(8.68,4738.13)_ = 56.96; *V* = 0.28		*F*_(8.69,4760.24)_ = 36.01; *V* = 0.37	
Medication			*n* = 247 (9.7% (s.e. = 0.64))^a^	
No need	92.3 (0.56)	94.4 (0.52)	20.6 (2.92)	42.5 (3.51)
Fully met need	2.7 (0.34)	3.3 (0.40)	28.2 (2.98)	34.5 (3.38)
Partially met need	1.4 (0.25)	1.1 (0.23)	13.8 (2.44)	10.9 (2.33)
Unmet need	3.6 (0.41)	1.2 (0.25)	37.4 (3.59)	12.1 (2.32)
	*F*_(8.45,4612.21)_ = 109.15; *V* = 0.42		*F*_(8.40,4602.76)_ = 16.04; *V* = 0.52	
Information			*n* = 689 (28.1% (s.e. = 1.09))^a^	
No need	81.1 (0.94)	85.1 (0.87)	32.8 (2.14)	46.8 (2.17)
Fully met need	11.4 (0.74)	7.5 (0.63)	40.4 (2.12)	26.8 (1.95)
Partially met need	3.5 (0.40)	2.9 (0.37)	12.5 (1.32)	10.3 (1.27)
Unmet need	4.0 (0.45)	4.5 (0.50)	14.3 (1.51)	16.1 (1.63)
	*F*_(8.34,4553.20)_ = 15.68; *V* = 0.15		*F*_(8.31,4555.76)_ = 31.16; *V* = 0.39	
Skill training			*n* = 583 (24.2% (s.e. = 0.98))^a^	
No need	85.7 (0.78)	86.5 (0.78)	40.5 (2.24)	44.3 (2.19)
Fully met need	6.1 (0.52)	4.3 (0.48)	25.4 (1.94)	17.6 (1.80)
Partially met need	2.2 (0.32)	1.6 (0.31)	9.2 (1.26)	6.8 (1.24)
Unmet need	6.0 (0.52)	7.6 (0.60)	24.9 (1.89)	31.3 (2.10)
	*F*_(8.57,4679.80)_ = 10.94; *V* = 0.12		*F*_(8.52,4670.45)_ = 32.99; *V* = 0.44	

*N*/*n*: unweighted number of respondents; s.e.: standard error; %: weighted percent; *V*: Cramer's *V* of 0.1, 0.3 and 0.5 represent small, medium and large strength of association.

a Of total sample.

Table 2 shows the agreement among adolescent-parent pairs on perceived need. Considering all four levels of perceived need, we found that agreement on perceived need for any type of help was fair. There was some variation in agreement across types of help needed – moderate for medication, fair for counselling and slight for skill training and information (Table 2). Inspection of cell frequencies of agreement showed, however, that majority of adolescent-parent pairs agreed on no need (online Supplementary eTable 4). When we excluded pairs that agreed that there was no need, agreement on the extent to which need was met decreased to slight for medication and to poor for any type of help and all other types of help (Table 2). In the sub-sample of adolescents with a probable disorder, results were generally similar except that agreement on the extent to which need for any type of help and for counselling was met was slight (online Supplementary eTable 5).

**Table 2.** Adolescent-parent agreement on perceived need

	Adolescent-parent agreement on perceived need (all levels) (*N* = 2310) %, Cohen's kappa (s.e.)	Adolescent-parent agreement on perceived need (excluding ‘no need’) %, Cohen's kappa (s.e.)
Any type of help	61.3, 0.25 (0.01)	20.0, −0.10 (0.02)
Counselling	71.5, 0.30 (0.01)	19.7, −0.10 (0.02)
Medication	92.0, 0.43 (0.01)	25.1, 0.01 (0.03)
Information	73.2, 0.15 (0.01)	10.2, −0.27 (0.02)
Skill training	76.6, 0.11 (0.01)	7.4, −0.34 (0.03)

unweighted %.

### Factors associated with patterns of adolescent-parent agreement on perceived need

Regression models examined associations of probable disorder and parental knowledge about adolescents' feelings with patterns of agreement, with ‘agreement that needs were fully met’ being the reference category. Having a probable disorder was positively associated with adolescent-parent agreement that needs were partially met or unmet, and, from the adolescents' perspective, negatively associated with adolescent-parent disagreement on perceived need. Little/no parental knowledge about adolescent's feelings was positively associated with agreement that needs were partially met or unmet and with disagreement between adolescent and parent (Table 3; full model in online Supplementary eTable 6); this was also found in the sub-sample of adolescents with a probable disorder (online Supplementary eTable 7). The *post hoc* analyses including probable externalising and probable internalising disorder separately showed that the association of probable disorder with patterns of agreement was mainly driven by probable internalising disorder. That is, having a probable internalising disorder was positively associated with agreement that needs were partially met or unmet, and negatively with both categories of adolescent-parent disagreement. Confidence intervals of associations for probable externalising disorder included zero and we can therefore not determine the direction of this effect (online Supplementary eTable 8).

**Table 3.** Fully adjusted^a^ multinomial logistic regressions of variables associated with patterns of agreement on perceived need (*n*  =  1119)

	Agreed that needs were fully met	Agreed that needs were partially met or unmet	Adolescent reported greater level of need than their parent	Parent reported greater level of need than the adolescent
	
	RRR (95% CI)			
Adolescents' probable disorder^b^				
Present	1.00	2.86 (1.46–5.61)	0.50 (0.30–0.82)	0.77 (0.47–1.24)
Parental knowledge about adolescents' feelings^c^				
Little/not at all	1.00	4.69 (2.38–9.28)	3.45 (2.16–5.51)	1.91 (1.19–3.04)

RRR: relative risk ratio; CI: confidence interval; *F*_(27,522)_ = 6.76.

a Estimates are adjusted for probable disorder or parental knowledge about adolescents' feelings, respectively, and sex, socio-economic (dis)advantage, remoteness, family type, parental education, parental psychopathology and family functioning.

b Reference category: absent.

c Reference category: ‘A lot/some’.

### Level of adolescent-parent agreement on barriers to care

**Fig. 2.**
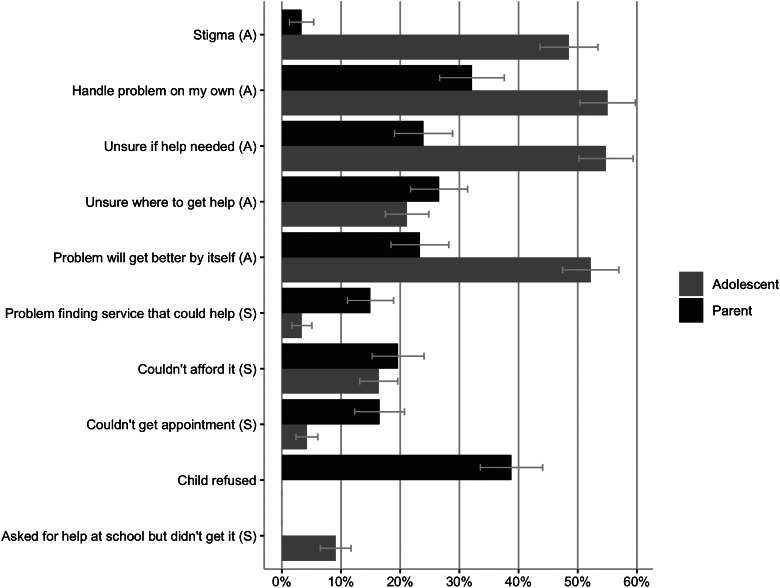
Barriers to care endorsed by adolescents (*n*  =  515) or parents (*n*  =  402). *Note:* A: attitudinal, S: structural. Each endorsed and denied barrier for both parents and adolescents adds up to 100%. Weighted percent (%) with 95%-confidence intervals (CIs). Differences in reported barriers are considered present if CIs of adolescent- and parent-reported barriers do not overlap.

Among the two sub-samples of adolescents *or* parents that identified an unmet or partially met need, adolescents reported attitudinal barriers most frequently. Comparing distributions of barriers among these two sub-samples, adolescents reported following barriers more frequently than their parents did (confidence intervals of estimates do no overlap (Schenker and Gentleman, 2001; Knol *et al*., 2011)): worried what others might think (stigma), wanting to handle the problem on my own, being unsure if help is needed and thinking that the problem will get better by itself. Parents most frequently reported that their child refused help and they reported following barriers more frequently than adolescents did: couldn't get an appointment when needed and having problems finding a service that could help (Fig. 2). Overall, attitudinal barriers were reported more frequently than structural barriers by both adolescents and parents (online Supplementary eTable 9). Among the smaller sub-sample where *both* adolescents and parents reported an unmet or partially met need, distributions of reported barriers were similar, but agreement was poor (*κ* = −0.03, s.e.  =  0.06) for reporting at least one attitudinal and slight (*κ* = 0.02, s.e.  =  0.09) for reporting at least one structural barrier (online Supplementary eTable 9).

## Discussion

This study had four key findings. First, almost half of either adolescents or parents identified a perceived need for adolescent mental health care. Need for any type of care was met in only a third and for specific types of help in a quarter to a third of adolescents. Second, adolescent-parent agreement on perceived need was higher regarding perceived need than regarding the extent to which perceived need was met. Third, the lack of communication between adolescents and parents, and for some adolescents, having a probable disorder increased the likelihood of unmet need. Fourth, adolescents and parents identified different barriers to care, and adolescent-parent agreement on barriers was low. In particular, attitudinal barriers appear to be important to adolescents, while the child refusing help appears to be important to parents.

Our study provides the first findings from a nationally representative sample of both adolescents' and parents' perspectives on perceived need for and barriers to adolescent mental health care. It includes individuals who may not meet criteria for a mental disorder; this is important because many adolescents who use mental health care might have subthreshold problems or other indicators of mental ill-health (Lawrence *et al*., 2015). However, the survey response-rate was modest, but declining response rates have been common in household survey for the last two decades (National Research Council, 2013) and the response rate for the YMM survey is very similar to that of comparable contemporary surveys (Vizard *et al*., 2018). The sample has been shown to be representative of the Australian adolescent population on major sociodemographic characteristics (Hafekost *et al*., 2016), but we cannot discount the possibility of other unmeasured biases related to perceived need. Parental knowledge about adolescent's feelings relied on a single item that was not tested for validity or reliability. The measure of probable disorder covered the past six months, different to the 12-months timeframe for perceived need, and it covered common mental disorders but not all; therefore, our findings might have not entirely captured the effect of a probable disorder on patterns of agreement. A limitation of the cross-sectional methodology is that some respondents might still have been receiving care at the time of interview; their level of perceived need may change when treatment is completed. Longitudinal studies are needed to resolve this problem. The barrier ‘worried what others might think/didn't want to talk to a stranger’ might capture different types of stigma, e.g. self-stigma and attitudes towards help-seeking. These should be assessed separately in future studies as they are differentially associated with help-seeking (Schnyder *et al*., 2017). Due to small cell sizes, we were not able to examine agreement on individual barriers.

Importantly, our findings suggest that there is a gap in the extent to which adolescent mental health care meets community needs that includes roughly equal proportions of adolescents (up to 20%) who needed help but did not receive it and adolescents who received help but not enough of it. Efforts to close this gap should take both groups into account. Perceived need for counselling, information and skill training seem to be more pronounced than the need for medication. Despite Australia's investment in improving access to psychological therapies, perceived need for this type of care for adolescents is not yet sufficiently met. This might be improved by ensuring that delivered treatment meets standards of minimally adequate care (Sawyer *et al*., 2018) and aligns with recommendations of evidence-based treatment guidelines (Crome and Baillie, 2016), educating clinicians in how to deliver patient-centred care and improving the provision of information about treatment (Gondek *et al*., 2017) and reducing barriers to care (Gulliver *et al*., 2010).

Our finding of low-adolescent-parent agreement regarding perceived need is similar to findings on agreement regarding mental health problems (Sourander *et al*., 1999; Fisher *et al*., 2006). A good therapeutic alliance – commonly including the bond between patient and therapist, and agreement about goals and tasks of therapy (Wampold *et al*., 1997) – might be beneficial for families to engage and retain in treatment (Gopalan *et al*., 2010). For adolescent mental health professionals, this alliance might be strengthened by aligning adolescents and parents needs at the beginning of and throughout treatment. One study of community-based adolescent outpatient service users suggested that concordance on perceived need for a counsellor or psychiatrist may be associated with higher numbers of appointments kept (Williams *et al*., 2011); this should be explored in general population studies with broader implications for policy and service development.

We proposed two potentially important factors for explaining why some needs for adolescent mental health care might not be met. Parental knowledge about adolescent's feelings, an indicator of lack of effective communication between adolescents and their parents, was associated with all groups of patterns of agreement on perceived need, differentiated the adolescent-parent pairs who agreed that needs had been fully meet from other groups. Australia's *headspace* ‘Fathers Campaign’ is an example of a public health intervention aimed at improving communication of mental health issues between fathers and sons. Our findings suggest that this approach could potentially be expanded to all adolescents and their families and should be evaluated to determine its effect on adolescent-parent agreement on perceived need.

Regarding probable disorder, there were two findings: (1) adolescents with a probable disorder were less likely than adolescents without a probable disorder to have their needs fully met. For healthcare providers, it might be more difficult to meet the needs of adolescents with a probable disorder as they might be more complex or require more intensive intervention than those of adolescents who do not meet the threshold for probable disorder. (2) Adolescents with a probable disorder were more likely to perceive the same level of need as perceived by their parents. This might be an encouraging finding as those adolescents might have a greater chance that their needs will be fully met because their needs are aligned with their parents'. *Post-hoc* specified analyses showed that these two findings were mainly driven by adolescents with a probable internalising disorder. This might be surprising, given evidence that parents and adolescents are more likely to agree on the presence of externalising than internalising problems (Rescorla *et al*., 2017). Our findings suggest that agreement on the problem area might not necessarily translate into agreement on perceived need for care. Future studies might investigate reasons for that.

Findings regarding the most frequent barriers to care concur with earlier studies (Jorm *et al*., 2007; Gulliver *et al*., 2010; Sheppard *et al*., 2018). Additionally, we showed that adolescent-parent agreement on barriers to care was low, indicating that campaigns directed towards adolescents and parents need to address different barriers. For adolescents, they might address attitudinal barriers such as stigma and mental health literacy (specifically, when to seek help). The implications of other barriers are less clear and require further examination. For example, self-reliance is a key barrier identified in earlier studies (e.g. Gulliver *et al*., 2010; Sheppard *et al*., 2018), but the reasons for this are unclear. Maybe it overshadows the importance of stigma (Jennings *et al*., 2015); reducing stigma might in turn reduce the desire for self-reliance. Alternatively, it might reflect a lack of understanding of the potential benefits of appropriate treatment (Jorm *et al*., 2006) and increasing mental health literacy might help. Thinking that the problem will get better by itself might seem a legitimate reason for not seeking help as spontaneous remission can sometimes occur (Beesdo-Baum *et al*., 2012), but longitudinal studies are needed to investigate whether those reporting this barrier actually get better without treatment. For parents, refusal of the child to receive help is a major barrier; future studies might examine whether the adolescents in these instances are those who did not perceive a need for care or who reported that their needs were not fully met.

## Conclusion

The mental healthcare system seems to meet needs for adolescent mental health care for one-quarter to one-third of adolescents and parents. Promoting conversation between adolescents and their parents might improve agreement between the two regarding perceived need and, in turn, provide opportunities to increase the likelihood that needs will be fully met. For adolescents, stigma has to be reduced and mental health literacy improved to reduce barriers to care. The ambiguity of other prominent barriers to adolescent mental health care calls for more research.

## Supplementary material

For supplementary material accompanying this paper visit http://dx.doi.org/10.1017/S2045796019000568. click here to view supplementary material

## Data

The Australian Data Archive (ADA) provided us with the YMM data. We are not able to directly share the YMM survey data but researchers in- and outside of Australia can apply for the data with the ADA. The data application form, documentations, and, ones ADA granted access, the data itself can be accessed here. Stata and R scripts that were used for statistical analyses in this publication can be found in the online Appendix (online Supplementary eMaterial 2).
