# Supplementary material for: Perceived need and barriers to adolescent mental health care: agreement between adolescents and their parents
Source: Epidemiol Psychiatr Sci. 2019 Sep 20;29:e60. doi: 10.1017/S2045796019000568 (PMC8061128; doi:10.1017/S2045796019000568)
Supplement: Supplementary file 1 [file S2045796019000568sup001.pdf]

Supplement to

Perceived Need and Barriers to Care in Adolescents:

Agreement Between Adolescents and Their Parents

Running head: Adolescent-parent agreement on perceived need and barriers

Schnyder, N.,[1,2] Lawrence, D.,[3] Panczak, R.,[4] Sawyer, M.G.,[5,6] Whiteford, H.A.,[1,2,7] Burgess, P.M.,[1,2] Harris, M.G.,[1,2]

[1] School of Public Health, The University of Queensland, Brisbane, Australia;

[2] Policy and Epidemiology Group, Queensland Centre for Mental Health Research, Brisbane, Australia;

[3] Graduate School of Education, The University of Western Australia, Perth, Australia;

[4] Queensland Centre for Population Research, School of Earth and Environmental Science, The University of Queensland, Brisbane, Australia;

[5] School of Medicine, University of Adelaide, Adelaide, SA, Australia;

[6] Research and Evaluation Unit, Women's and Children's Health Network, Adelaide, SA, Australia;

[7] Institute for Health Metrics and Evaluation, University of Washington, Seattle, Washington, USA.

Content

eTable 1 Classification of adolescent-parent agreement on overall perceived need (N=2,310).

eTable 2 Sample characteristics of adolescents aged 13-17.

eMaterial 1 Additional measures, how they were classified and used.

eTable 3 Distribution and comparison of past 12 months overall perceived need and types of help needed among sub-sample of adolescents (aged 13-17) with a probable disorder and their parents.

eTable 4 Details of agreement on overall perceived need and each type of help needed.

eTable 5 Adolescent-parent agreement on perceived need among sub-sample of adolescents with a probable disorder.

eTable 6 All results of multinomial logistic regressions of variables associated with patterns of agreement on perceived need (n=1,119).

eTable 7 Multinomial logistic regressions of variables associated with patterns of agreement on perceived need among those with a probable disorder (n=325).

eTable 8 All results of multinomial logistic regressions of variables associated with patterns of agreement on perceived need; internalising and externalising disorder separately (n=1,119).

eTable 9 Barriers to care among sub-sample where either the adolescent or parent identified an unmet or partially met need.

eMaterial 2 Stata and R scripts.

eTable 1 Classification of adolescent-parent agreement on overall perceived need (N=2,310).

		Adolescents			
		Fully met need	Partially met need	Unmet need	No need
Parents	Fully need	A	C	C	D
	Partially met need	D	B	C	D
	Unmet need	D	D	B	D
	No need	C	C	C	E

Note N/n: unweighted number of respondents with 'N' representing total population and 'n' representing sub-population.

The categories of the dependent variables are (see also table below A, B, C, D):

- A: Adolescents and parents agree on fully met need (reference group, n=131),
- B: Adolescents and parents agree on either partially met or unmet need (n=93),
- C: Adolescents perceived a greater level of need than their parents (n=488),
- D: Parents perceived a greater level of need than their child (n=407),
- E: Adolescents and parents agree on having no need (n=1,191).

eMaterial 1 Additional measures, how they were used in this study.

What was measured	Measure (questionnaire or questions)	How we used the measure (categories of measures are indicated in *italic*)	Regression used as:
Adolescents' probable disorder	Sum score of four subscales of the 'Strengths and Difficulties Questionnaire' (SDQ) (Goodman 1997, 2001) for total difficulties: emotional symptoms, hyperactivity, conduct problems, and peer problems. Total difficulties score ranges from 0-40 with higher scores indicating more problems and with 80% of adolescents in the community having normal levels of difficulties and 10% either borderline or abnormal levels. Abnormal levels are referred to as an indication for having a probable disorder (Goodman 2001). For total difficulties in this study of adolescents aged 13-17: Cronbach's $\alpha_{(parent\ report)}$=0.72; Cronbach's $\alpha_{(adolescent\ report)}$=0.70.	We defined the *presence* of a probable disorder as either adolescents or parents reporting total difficulties on an abnormal level, while we defined the *absence* of probable disorder as both adolescents and parents reporting total difficulties on a normal or borderline level. This allowed us to consider both adolescents' and parents' perceptions about adolescent mental health problems because their agreement on symptoms is typically modest (Rescorla *et al.* 2013).	Independent variable
Adolescents' probable internalising disorder or probable externalising disorder	SDQ subscales can be summarised into internalising (emotional and peer subscale) and externalising (conduct and hyperactivity subscales) problems (Goodman *et al.* 2010). Each of the subscale ranges from 0-10 with higher scores indicating more problems and with 80% of adolescents in the community having normal levels of problems in respective subscale and 10% either borderline or abnormal levels. Abnormal levels are used as an indication for having a probable emotional, conduct or hyperactivity disorder (Goodman 2001). For probable internalising disorder in this study: Cronbach's $\alpha_{(parent\ report)}$=0.62; Cronbach's $\alpha_{(adolescent\ report)}$=0.60. For probable externalising disorder in this study Cronbach's $\alpha_{(parent\ report)}$=0.70; Cronbach's $\alpha_{(adolescent\ report)}$=0.68.	First, separately for adolescents and parents, probable internalising disorders were defined as reporting abnormal levels on the subscales emotional and/or peer problems; probable externalising disorders as reporting abnormal levels on conduct and/or hyperactivity problems.<br>Second, we defined the *presence* of a probable internalising disorder as either adolescents *or* parents reporting it on an abnormal level, while we defined the *absence* of probable internalising disorder as both adolescents and parents reporting internalising disorders on a normal or borderline level. We proceeded accordingly for the presence and absence of externalising disorders. This was done before by (Downs *et al.* 2013).	Independent variable

eMaterial 1 *continued*

**What was measured**	**Measure (questionnaire or questions)**	**How we used the measure** (categories of measures are indicated in *italic*)	**Regression used as:**
Parents' knowledge about adolescents' feelings	Question: 'How much do your parents know about how you are feeling?'<br>Answered on a 4-point likert scale from 'a lot' to 'not at all'.	We collapsed four categories into two ('*a lot/some*' and '*a little/not at all*').	Independent variable
General family functioning	Reliable and valid six items version (Boterhoven De Haan *et al.* 1985) of the McMaster Family Functioning scale (Epstein *et al.* 1983). Items are rated on a 4-point likert scale, answers are summed up and divided by the number of items to receive a score between 1-6 (Miller *et al.* 1985). In this study including parents with adolescents aged 13-17, Cronbach's α=0.87.	Used as proposed by Miller and colleagues (1985): score of >2 indicate *healthy* and ≤2 *unhealthy family* functioning.	Control variable
Parental psychopathology	(1) Parents levels of psychological distress in the past four weeks was assessed with the 10-item Kessler Psychological Distress Scale (Kessler *et al.* 2003). In this study including parents with adolescents aged 13-17, Cronbach's α=0.90.<br>(2) Question for lifetime disorder: 'Have you ever been told by a doctor or mental health professional that you have any of these problems?':<br>• Panic attacks<br>• Post-traumatic stress disorder (PTSD)<br>• Obsessive-compulsive disorder (OCD)<br>• Any other anxiety problems<br>• Depression<br>• Attention Deficit Disorder (ADD) / Attention Deficit Hyperactivity Disorder (ADHD)<br>• Schizophrenia<br>• Bipolar disorder or any other psychosis<br>• Alcohol or drug dependence<br>• Other mental health problem	Based on an earlier YMM study (Johnson *et al.* 2018), parental psychopathology was considered to be *present* if parents reported a lifetime diagnosis of a mental disorder and/or (very) high levels of psychological distress in the past four weeks according to the 10-item Kessler Psychological Distress Scale. Otherwise, parental psychopathology was considered to be *absent*.	Control variable

eMaterial 1 *continued*

What was measured	Measure (questionnaire or questions)	How we used the measure (categories of measures are indicated in *italic*)	Regression used as:
Family type	The Young Minds Matter (YMM) survey assessed family type according to the Australian Bureau of Statistic's (ABS) definition of family blending as living with: (1) two biological parents (nuclear family), (2) other two parent family, (3) sole parent family), (4) other.	We collapsed these four categories into two: (1) *family with two biological parents* or (2) other *family type*.	Control variable
Remoteness	YMM assessed remoteness according to the Australian Statistical Geography Standard-Remoteness Area (ASGS-RA) provided by the ABS. It is a geographical classification which defines place of residence/location in terms of remoteness. Remoteness is categorised as: (1) Major cities of Australia, (2) inner regional Australia, (3) outer regional Australia, (4) remote Australia.	We collapsed remoteness into the two categories '*major cities*' (1) and '*regional and remote areas*' (2-4).	Control variable
Socio-economic advantage and disadvantage (IRSAD)	YMM assessed socio-economic advantage and disadvantage according to the Socio-Economic Index for Areas (SEIFA index) of the ABS. The SEIFA index defines socio-economic advantage and disadvantage according to the place of residence rather than to the individuals' actual status of socio-economic advantage and disadvantage. The SEIFA index contains the 'index of relative socio-economic advantage and disadvantage' (IRSAD) which was used in this study. IRSAD can be divided into quintiles with lowest quintile (most disadvantaged) to second quintile, to third quintile, to fourth quintile and to highest quintile (most advantaged).	We collapsed IRSAD quintiles into *advantaged* (highest and fourth quintile) and *disadvantaged* (lowest, second and third quintile).	Control variable
Parental education	Parent/primary carer was asked: 'What is the level of the highest post-school qualification that you have completed?':<br>• Postgraduate degree, graduate diploma or graduate certificate<br>• Bachelor degree, advanced diploma or diploma<br>• Certificate III/IV, certificate I/II<br>• Certificate not further defined<br>• No non-school qualification<br>• Level not determined	Highest level of parental education was collapsed in two categories '*bachelor degree or higher*' and *'diploma or certificate III/IV or lower*'.	Control variable
Adolescents' sex	Female or male	*Female* or *male*	Control variable

eTable 2 Sample characteristics of adolescents aged 13-17.

	Total sample (N=2,310)	Sub-sample[a] (n=1,119)
	%(SE)	*%(SE)*
Sex[b]		
Female	48.7(1.16)	46.2(1.68)
Male	51.3(1.16)	53.8(1.68)
Index of relative socio-economic disadvantage (IRSAD)[b]		
Advantaged	45.4(2.58)	43.7(2.78)
Disadvantaged	54.6(2.58)	56.3(2.78)
Remoteness[b]		
Major cities of Australia	64.0(2.51)	65.3(2.72)
Regional or remote areas in Australia	36.0(2.51)	34.7(2.72)
Family type[b]		
Family with two biological parents	59.9(1.27)	52.6(1.76)
Other family type	40.1(1.27)	47.4(1.76)
Parental education[b]		
Bachelor degree or higher	38.0(1.41)	39.1(1.77)
Diploma or certificate III/IV or lower	62.0(1.41)	60.9(1.77)
Parental psychopathology[b]		
Lifetime diagnosis or *current high/very high psychological distress*	41.6(1.17)	49.3(1.78)
None	58.4(1.17)	50.7(1.78)
Family functioning[b]		
Healthy level of functioning	95.9(0.45)	93.6(0.83)
Unhealthy level of functioning	4.1(0.45)	6.4(0.83)
Adolescents' probable disorder[b]		
Present	16.7(0.93)	29.4(1.61)
Absent	83.3(0.93)	70.6(1.61)
Adolescents' probable externalising disorder[b]		
Present	27.0(1.10)	39.4(1.73)
Absent	73.0(1.10)	60.6(1.73)
Adolescents' probable internalising disorder[b]		
Present	31.4(1.11)	46.9(1.68)
Absent	68.6(1.11)	53.1(1.68
Parental knowledge about adolescents' feelings[b]		
A Lot/some	68.7(1.11)	58.7(1.67)
Little/not at all	31.3(1.11)	41.4(1.67)

[a] *either* adolescent *or* parent identified a perceived need for any type of care; [b] for details on measures and their use see eMaterial 1.

Note N/n: unweighted number of participants; SE: standard error; %: weighted percent.

eTable 3 Distribution and comparison of past 12 months overall perceived need and types of help needed among sub-sample of adolescents (aged 13-17) with a probable disorder and their parents.

Level of perceived need, by type of help	*Total sample with a probable disorder (n=390)*		*Sub-samples,* either *adolescent* or *parent identified a perceived need*	
	Adolescents, *%(SE)*	Parents, *%(SE)*	Adolescents, *%(SE)*	Parents, *%(SE)*
Any type of help			n=325 (82.2%(SE=2.11))[a]	
No need	39.9(2.85)	31.6(2.52)	27.0(2.98)	16.8(2.21)
Fully met need	20.9(2.15)	24.6(2.36)	25.4(2.55)	29.9(2.78)
Partially met need	27.4(2.44)	28.6(2.46)	33.3(2.87)	34.8(2.89)
Unmet need	11.8(1.82)	15.2(2.05)	14.3(2.17)	18.5(2.42)
	$F_{(8.69,4761.31)}$=7.01; V=0.25		$F_{(8.67,4752.12)}$=8.23; V=0.30	
Counselling			n=290 (73.6%(SE=2.44))[a]	
No need	53.7(2.83)	37.3(2.68)	37.2(3.24)	14.8(2.23)
Fully met need	21.9(2.62)	25.7(2.49)	29.7(2.91)	34.9(3.17)
Partially met need	14.9(1.96)	18.9(2.18)	20.2(2.61)	25.7(2.81)
Unmet need	9.5(1.57)	18.1(2.16)	12.9(2.07)	24.6(2.83)
	$F_{(8.51,4664.31)}$=9.68; V=0.28		$F_{(8.50,4657.53)}$=10.45; V=0.34	
Medication			n=158 (37.3%(SE=2.69))[a]	
No need	69.8(2.48)	76.0(2.36)	19.0(3.29)	35.6(4.20)
Fully met need	11.3(1.60)	12.9(1.88)	30.4(3.72)	34.7(4.30)
Partially met need	6.6(1.29)	5.1(1.2)	17.6(3.18)	13.7(3.15)
Unmet need	12.3(1.80)	5.9(1.23)	33.1(4.28)	16.0(3.01)
	$F_{(8.46,4634.30)}$=20.28; V=0.44		$F_{(8.37,4586.47)}$=11.99; V=0.55	
Information			n=248 (62.1%(SE=2.63))[a]	
No need	60.4(2.83)	55.4(2.82)	36.3(3.57)	28.2(3.11)
Fully met need	19.1(2.15)	19.1(2.29)	30.8(3.26)	30.8(3.38)
Partially met need	10.0(1.59)	10.8(1.73)	16.1(2.41)	17.4(2.68)
Unmet need	10.5(2.83)	14.6(1.97)	16.8(2.70)	23.6(2.98)
	$F_{(8.30,4549.50)}$=5.35; V=0.21		$F_{(8.26,4527.60)}$=9.32; V=0.35	
Skill training			n=209 (52.9%(SE=2.77))[a]	
No need	70.8(2.33)	65.4(2.53)	44.8(3.50)	34.6(3.35)
Fully met need	10.0(1.64)	7.3(1.40)	19.0(2.95)	13.8(2.56)
Partially met need	7.1(1.29)	5.1(1.31)	13.4(2.43)	9.6(2.42)
Unmet need	12.1(1.68)	22.2(2.22)	22.8(2.89)	42.0(3.55)
	$F_{(8.28,4538.78)}$=3.31; V=0.16		$F_{(8.33,4567.03)}$=13.37; V=0.43	

[a] of total sample with a probable disorder.
Note N/n: unweighted number of respondents; SE: standard error; %: weighted percent; V: Cramer's V of 0.1, 0.3, and 0.5 represent small, medium, and large strength of association.

eTable 4 Details of agreement on overall perceived need and each type of help needed between adolescents (aged 13-17) and their parents

			Parents			
			Overall perceived need, *n (%)*			
			Fully met need	Partially met need	Unmet need	No need
Adolescents	Overall perceived Need, *n (%)*	Fully need	131 (5.7)	65 (2.8)	25 (1.1)	196 (8.5)
		Partially met need	59 (2.5)	70 (3.0)	17 (0.7)	83 (3.6)
		Unmet need	25 (1.1)	13 (0.6)	23 (1.0)	112 (4.8)
		No need	125 (5.4)	62 (2.7)	113 (4.9)	**1,191 (51.6)**
			Need for counselling, *n (%)*			
			Fully met need	Partially met need	Unmet need	No need
	Need for counselling, *n (%)*	Fully need	113 (4.9)	46 (2.0)	17 (3.5)	81 (3.5)
		Partially met need	44 (1.9)	27 (1.2)	9 (0.4)	32 (1.4)
		Unmet need	16 (0.7)	11 (0.5)	22 (1.0)	104 (4.5)
		No need	112 (4.9)	56 (2.4)	131 (5.7)	**1,489 (64.5)**
			Need for medication, *n (%)*			
			Fully met need	Partially met need	Unmet need	No need
	Need for medication, *n (%)*	Fully need	47 (2.0)	9 (0.4)	0 (0.0)	18 (0.8)
		Partially met need	17 (0.7)	10 (0.4)	0 (0.0)	6 (0.3)
		Unmet need	4 (0.2)	1 (0.1)	5 (0.2)	83 (3.6)
		No need	18 (0.8)	5 (0.2)	24 (1.0)	**2,063 (89.3)**
			Need for information, *n (%)*			
			Fully met need	Partially met need	Unmet need	No need
	Need for information, *n (%)*	Fully need	50 (2.2)	14 (0.6)	17 (0.7)	208 (9.0)
		Partially met need	20 (0.9)	11 (0.5)	6 (0.2)	50 (2.1)
		Unmet need	5 (0.2)	7 (0.4)	9 (0.4)	81 (3.5)
		No need	101 (4.4)	36 (1.5)	74 (3.2)	**1,621 (70.2)**
			Need for skill training, *n (%)*			
			Fully met need	Partially met need	Unmet need	No need
	Need for skill training, *n (%)*	Fully need	19 (0.8)	2 (0.1)	18 (0.8)	109 (4.7)
		Partially met need	2 (0.1)	5 (0.2)	18 (0.8)	31 (1.3)
		Unmet need	9 (0.4)	4 (0.2)	19 (0.8)	126 (5.5)
		No need	67 (2.9)	22 (0.9)	132 (5.7)	**1,729 (74.8)**

Note n: unweighted number of respondents; %: unweighted percent; cells shaded in blue mark those adolescents and parents that agree, cell frequencies in **bold** mark the ones with the largest agreement for overall perceived need and for each of the types of help.

eTable 5. Adolescent-parent agreement on perceived need among sub-sample of adolescents with a probable disorder.

	Agreement on perceived need (all levels) (n=390)<br>*%, Cohen's kappa(SE)*	Agreement on perceived need (excluding 'no need') (n=325)<br>*%, Cohen's kappa(SE)*
Any type of help	41.8, 0.20(0.03)	30.2, 0.05(0.03)
Counselling	44.9, 0.22(0.03)	25.9, 0.01(0.03)
Medication	71.3, 0.39(0.03)	29.1, 0.05(0.04)
Information	48.7, 0.16(0.03)	19.4, -0.10(0.04)
Skill training	51.5, 0.05(0.03)	9.6, -0.27(0.04)

Note unweighted %; kappa of <0.00, 0.00-0.20, 0.21-0.40, 0.41-0.60, 0.61-0.80, and 0.81-1.00 are considered to represent poor, slight, fair, moderate, substantial, and almost perfect agreement (Landis & Koch 1977).

eTable 6 All results of multinomial logistic regressions of variables associated with patterns of agreement on perceived need (n=1,119).

	Agreed that needs were fully met		Agreed that needs were partially met or unmet		Adolescent reported greater level of need than their parent		Parent reported greater level of need than the adolescent	
	Unadjusted	Fully adjusted [a]	Unadjusted	Fully adjusted [a]	Unadjusted	Fully adjusted [a]	Unadjusted	Fully adjusted [a]
	RRR	RRR	RRR (95%-CI)	RRR (95%-CI)	RRR (95%-CI)	RRR (95%-CI)	RRR (95%-CI)	RRR (95%-CI)
Adolescents' probable disorder								
Present	1.00	1.00	3.77(2.02-7.04)	2.86(1.46-5.61)	0.61(0.38-0.96)	0.50(0.30-0.82)	1.01(0.64-1.60)	0.77(0.47-1.24)
Parental knowledge about adolescents' feelings								
Little/not at all	1.00	1.00	5.36(2.85-10.09)	4.69(2.38-9.28)	3.12(1.98-4.90)	3.45(2.16-5.51)	1.83(1.16-2.90)	1.91(1.19-3.04)
Sex								
Female	1.00	1.00	1.93(1.04-3.58)	1.87(1.00-3.48)	1.28(0.84-1.95)	1.23(0.80-1.89)	0.61(0.40-0.95)	0.60(0.38-0.93)
Index of relative socio-economic advantage and disadvantage (IRSAD)								
Disadvantaged	1.00	1.00	0.57(0.32-1.03)	0.48(0.25-0.94)	0.71(0.47-1.07)	0.72(0.46-1.13)	0.96(0.62-1.49)	0.78(0.49-1.25)
Remoteness								
Regional or remote area	1.00	1.00	0.91(0.50-1.66)	0.96(0.50-1.85)	0.91(0.57-1.44)	0.95(0.58-1.54)	1.35(0.85-2.13)	1.23(0.76-1.99)
Family type								
Living with two biological parents	1.00	1.00	1.63(0.94-2.83)	2.13(1.16-3.90)	1.92(1.25-2.95)	1.81(1.15-2.82)	1.18(0.76-1.85)	1.39(0.86-2.14)
Parental education								
Diploma or certificate III/IV or lower	1.00	1.00	1.31(0.73-2.37)	1.33(0.70-2.55)	1.06(0.70-1.60)	1.36(0.88-2.11)	1.97(1.27-3.04)	2.13(1.35-3.38)
Parental psychopathology								
Current symptoms or lifetime disorder	1.00	1.00	1.26(0.72-2.22)	1.39(0.78-2.48)	0.61 0.40-0.93)	0.75(0.49-1.14)	1.15(0.75-1.77)	1.17(0.78-1.82)
Family functioning								
Unhealthy level of functioning	1.00	1.00	5.19(1.28-21.03)	3.81(0.94-15.40)	2.13(0.56-8.11)	2.63(0.70-9.93)	3.26(0.90-11.78)	2.94(0.80-10.81)

[a] All variables are added simultaneously to the model.

Reference categories of independent/control variables are: male sex, advantaged IRSAD, major cities (remoteness), living in another family constellation (family type), bachelor degree or higher (parental education), normal family functioning, adolescent probable disorder absent, parent has 'a lot/some' knowledge of adolescent feelings.

Note RRR: relative risk ratio; CI: confidence interval; $F_{(27,522)}$=6.76.

eTable 7 Multinomial logistic regressions of variables associated with patterns of agreement on perceived need among those with a probable disorder (n=325).

	Agreed that needs were fully met		Agreed that needs were partially met or unmet		Adolescent reported greater level of need than their parent		Parent reported greater level of need than the adolescent	
	Unadjusted	Fully adjusted [a]	Unadjusted	Fully adjusted [a]	Unadjusted	Fully adjusted [a]	Unadjusted	Fully adjusted [a]
	RRR	RRR	RRR (95%-CI)	RRR (95%-CI)	RRR (95%-CI)	RRR (95%-CI)	RRR (95%-CI)	RRR (95%-CI)
Parental knowledge about adolescents' feelings								
Little/not at all	1.00	1.00	3.76 (1.86-7.63)	2.90 (1.19-7.12)	3.75 (2.13-6.61)	3.88 (1.74-8.68)	1.93 (1.12-3.34)	2.39 (1.14-5.03)
Sex								
Female	1.00	1.00	1.89 (0.76-4.69)	0.63 (0.25-4.45)	1.60 (0.76-3.39)	1.50 (0.72-3.15)	0.53 (0.24-1.16)	0.53 (0.24-1.13)
Index of relative socio-economic advantage and disadvantage (IRSAD)								
Disadvantaged	1.00	1.00	0.71 (0.30-1.69)	0.65 (0.31-1.59)	0.88 (0.41-1.92)	0.76 (0.33-1.77)	1.98 (0.91-4.28)	1.47 (0.65-3.34)
Remoteness								
Regional or remote area	1.00	1.00	1.32 (0.55-3.16)	1.18 (0.46-3.06)	1.20 (0.52-2.76)	1.10 (0.45-2.67)	1.52 (0.71-3.23)	1.23 (0.54-2.77)
Family type								
Family with two biological parents	1.00	1.00	1.89 (0.80-4.45)	2.25 (0.89-5.70)	1.32 (0.60-2.90)	1.39 (0.55-3.51)	0.89 (0.41-1.91)	1.17 (0.50-2.71)
Parental education								
Diploma or certificate III/IV or lower	1.00	1.00	1.19 (0.45-3.18)	1.69 (0.58-4.99)	1.66 (0.73-3.76)	1.86 (0.75-4.56)	2.48 (1.06-5.84)	2.24 (0.88-5.75)
Parental psychopathology								
Current symptoms or lifetime disorder	1.00	1.00	1.31 (0.54-3.19)	1.69 (0.68-4.20)	0.59 (0.26-1.33)	0.68 (0.30-1.54)	1.49 (0.65-3.38)	1.51 (0.65-3.49)
Family functioning								
Unhealthy level of functioning	1.00	1.00	2.50 (0.44-14.25)	2.51 (0.43-14.58)	2.09 (0.30-11.05)	2.63 (0.47-14.60)	2.49 (0.49-12.65)	2.49 (0.42-14.87)

[a] All variables are added simultaneously to the model.

Reference categories of independent/control variables are: male sex, advantaged IRSAD, major cities (remoteness), living in another family constellation (family type), bachelor degree or higher (parental education), normal family functioning, parent has 'a lot/some' knowledge of adolescent feelings. The presence of a probable disorder was defined as either adolescents or parents reporting total difficulties (SDQ) on an abnormal level.

Note RRR: relative risk ratio; CI: confidence interval; $F_{(24,\ 525)}$=2.43.

eTable 8 All results of multinomial logistic regressions of variables associated with patterns of agreement on perceived need; internalising and externalising disorder separately (n=1,119).

	Agreed that needs were fully met		Agreed that needs were partially met or unmet		Adolescent reported greater level of need than their parent		Parent reported greater level of need than the adolescent	
	Unadjusted	Fully adjusted [a]	Unadjusted	Fully adjusted [a]	Unadjusted	Fully adjusted [a]	Unadjusted	Fully adjusted [a]
	RRR	RRR	RRR (95%-CI)	RRR (95%-CI)	RRR (95%-CI)	RRR (95%-CI)	RRR (95%-CI)	RRR (95%-CI)
Adolescents' probable externalising disorder								
Present	1.00	1.00	1.99(1.07-3.71)	1.67(0.84-3.29)	0.63(0.41-0.97)	0.68(0.43-1.08)	1.23(0.79-1.92)	1.16(0.73-1.84)
Adolescents' probable internalising disorder								
Present	1.00	1.00	3.03(1.59-5.78)	2.07(1.06-4.03)	0.50(0.33-0.77)	0.45(0.29-0.72)	0.67(0.44-1.03)	0.57(0.36-0.91)
Parental knowledge about adolescents' feelings								
Little/not at all	1.00	1.00	5.36(2.85-10.09)	4.76(2.48-9.15)	3.12(1.98-4.90)	3.61(2.26-5.79)	1.83(1.16-2.90)	1.91(1.19-3.04)
Sex								
Female	1.00	1.00	1.93(1.04-3.58)	1.84(0.99-3.43)	1.28(0.84-1.95)	1.31(0.86-2.02)	0.61(0.40-0.95)	0.66(0.43-1.03)
Index of relative socio-economic advantage and disadvantage (IRSAD)								
Disadvantaged	1.00	1.00	0.57(0.32-1.03)	0.47(0.25-0.91)	0.71(0.47-1.07)	0.74(0.48-1.16)	0.96(0.62-1.49)	0.79(0.50-1.26)
Remoteness								
Regional or remote area	1.00	1.00	0.91(0.50-1.66)	0.97(0.51-1.84)	0.91(0.57-1.44)	0.95(0.59-1.55)	1.35(0.85-2.13)	1.21(0.75-1.96)
Family type								
Living with two biological parents	1.00	1.00	1.63(0.94-2.83)	1.97(1.09-3.54)	1.92(1.25-2.95)	1.69(1.08-2.64)	1.18(0.76-1.85)	1.32(0.84-2.07)
Parental education								
Diploma or certificate III/IV or lower	1.00	1.00	1.31(0.73-2.37)	1.40(0.74-2.66)	1.06(0.70-1.60)	1.37(0.89-2.11)	1.97(1.27-3.04)	2.18(1.38-3.44)
Parental psychopathology								
Current symptoms or lifetime disorder	1.00	1.00	1.26(0.72-2.22)	1.36(0.76-2.43)	0.61 0.40-0.93)	0.78(0.51-1.19)	1.15(0.75-1.77)	1.21(0.78-1.87)
Family functioning								
Unhealthy level of functioning	1.00	1.00	5.19(1.28-21.03)	4.00(0.96-16.66)	2.13(0.56-8.11)	2.87(0.77-10.77)	3.26(0.90-11.78)	3.09(0.85-11.25)

[a] All variables are added simultaneously to the model.
Reference categories of independent/control variables are: male sex, advantaged IRSAD, major cities (remoteness), living in another family constellation (family type), bachelor degree or higher (parental education), normal family functioning, adolescent probable disorder absent, parent has 'a lot/some' knowledge of adolescent feelings.
Note RRR: relative risk ratio; CI: confidence interval; $F_{(30,519)}$=5.78.

Table 9 Barriers to care among sub-sample where either the adolescent or parent identified an unmet or partially met need

	Subsamples[a]		*Both* adolescents *and* parents have either a fully or partially unmet need (n=123)		
	Barriers endorsed by adolescents (n=515), *% (SE)*	Barriers endorsed by parents (n=402), *% (SE)*	Barriers endorsed by adolescents, *% (SE)*	Barriers endorsed by parents, *% (SE)*	Adolescent-parent agreement, *%, Cohen's kappa (SE)*
Any attitudinal barriers [b]	86.8 (1.75)	85.0 (1.82)	88.2 (3.37)	85.7 (3.15)	56.2, -0.03 (0.06)
afraid what others might think	48.5 (2.49)	3.3 (1.02)	50.3 (5.26)	3.3 (2.04)	-
self-reliance	55.1 (2.39)	32.5 (2.78)	52.2 (4.97)	27.5 (5.17)	-
unsure if help needed	54.8 (2.34)	24.9 (2.48)	53.4 (4.79)	20.8 (4.10)	-
unsure where to get help	21.2 (1.87)	27.6 (2.42)	25.5 (4.57)	27.7 (4.30)	-
problem will get better by itself	52.2 (2.43)	23.8 (2.48)	63.4 (4.77)	26.2 (4.44)	-
child refused	-	38.7 (2.69)	-	42.3 (5.11)	-
Any structural barriers [c]	25.3 (1.91)	33.0 (2.60)	40.3 (4.88)	36.6 (4.56)	53.3, 0.02 (0.09)
problem finding service that could help	3.4 (0.87)	15.0 (1.97)	9.7 (3.10)	19.1 (4.00)	-
couldn't afford it	16.4 (1.63)	20.0 (2.27)	23.5 (4.18)	19.9 (3.84)	-
couldn't get appointment	4.2 (0.94)	16.7 (2.09)	7.7 (2.56)	23.6 (4.04)	-
asked for help at school but didn't get any	9.1 (1.33)	-	15.2 (3.25)	-	-

[a] adolescents *or* parents reported barriers to care; [b] at least one attitudinal barrier was endorsed; [c] at least one structural barrier was endorsed.

Note n: unweighted number of respondents in sub-population; %: weighted percent; SE: standard error; percent in cells do not add up to 100% because naming multiple barriers was possible; kappa of <0.00, 0.00-0.20, 0.21-0.40, 0.41-0.60, 0.61-0.80, and 0.81-1.00 are considered to represent poor, slight, fair, moderate, substantial, and almost perfect agreement (Landis & Koch 1977).

Additional References

Boterhoven De Haan K, Hafekost J, Lawrence D, Sawyer MG, Zubrick SR (1985). Reliability and Validity of a Short Version of the General Functioning Subscale of the McMaster Family Assessment Device. *Family Process* **54**, 116–123.

Downs JM, Cullen AE, Barragan M, Laurens KR (2013). Persisting psychotic-like experiences are associated with both externalising and internalising psychopathology in a longitudinal general population child cohort. . Elsevier B.V. *Schizophrenia Research* **144**, 99–104.

Epstein NB, Baldwin LM, Bishop DS (1983). The McMaster Family Assessment Device. *Journal of Marital and Family Therapy* **9**, 171–180.

Goodman A, Lamping DL, Ploubidis GB (2010). When to use broader internalising and externalising subscales instead of the hypothesised five subscales on the strengths and difficulties questionnaire (SDQ): Data from british parents, teachers and children. *Journal of Abnormal Child Psychology* **38**, 1179–1191.

Goodman R (1997). The Strengths and Difficulties Questionnaire: A Research Note. *Journal of Child Psychology and Psychiatry* **38**, 581–586.

Goodman R (2001). Psychometric properties of the Strengths and Difficulties Questionnaire in 3-year-old preschoolers. *Journal of the American Academy of Child and Adolescent Psychiatry* **54**, 282–291.

Johnson SE, Lawrence D, Sawyer M, Zubrick SR (2018). Mental disorders in Australian 4- to 17- year olds: Parent-reported need for help. *Australian and New Zealand Journal of Psychiatry* **52**, 149–162.

Kessler R. C, Barker PR, Colpe LJ, Epstein JF, Gfroerer JC, Hiripi E, Howes MJ, Normand S-LT, Manderscheid RW, Walters EE, Zaslavsky AM (2003). Screening for Serious Mental Illness in the General Population. *Archives of general psychiatry* **60**, 184–189.

Landis JR, Koch GG (1977). The Measurement of Observer Agreement for Categorical Data. *Biometrics* **33**, 159–174.

Miller IW, Epstein NB, Bishop DS, Keitner GI (1985). The McMaster Family Assessment Device: Reliability and Validity. *Journal of Marital and Family Therapy* **11**, 345–356.

Rescorla LA, Ginzburg S, Achenbach TM, Ivanova MY, Almqvist F, Begovac I, Bilenberg N, Bird H, Chahed M, Dobrean A, Döpfner M, Erol N, Hannesdottir H, Kanbayashi Y, Lambert MC, Leung PWL, Minaei A, Novik TS, Oh KJ, Petot D, Petot JM, Pomalima R, Rudan V, Sawyer M, Simsek Z, Steinhausen HC, Valverde J, van der Ende J, Weintraub S, Metzke CW, Wolanczyk T, Zhang EY, Zukauskiene R, Verhulst FC (2013). Cross-Informant Agreement Between Parent-Reported and Adolescent Self-Reported Problems in 25 Societies. *Journal of Clinical Child and Adolescent Psychology* **42**, 262–273.

eMaterial 2 Stata and R scripts.

```
* ***********************************************************
* ***********************************************************
* ***********************************************************
* Young Minds Matter 01
* Analysis for perceived needs of adolescents and their parents and relationship between the two

* Author: Nina
* Date: September/October 2018
* ***********************************************************
* ***********************************************************
* ***********************************************************

/* explanation to data set
1.  Not needed variables were deleted from both data sets (youth and parent).
2.  Youth and parent YMM data sets were merged 1:1 according to variable householdID.
3.  Not all adolescents participated in self report. Variable yint11 was used to identify responders and delete all non-responders from data set.
--> non-responders: 13-17 year olds n=290, 11-12 year olds n=77 (total n=367)
4.  In surveys where weights and clustering refer to youth >= 11 years, 11-12 year olds can not just be deleted from data set.
--> Define subpopulation 'adolescents' for analyses.
*/

di in red c(os)

if "`c(os)'" == "MacOSX" {

        cd "/Users/admin/research/publications/08_YMM_PerceivedNeedAndBarriersToCare/"

}

* data set that will be used for analyses (excluding non-responder adolescents n=290)
use     Data/YMM2/AdolescentsParents_complete, clear

numlabel, add

* ***********************************************************
* ***********************************************************
* eFigure 1 response rate
* use variable yint11 to determine response rate of adolescents aged 13-17
use     Data/YMM2/cleaned_merged/YouthParents_merged, clear

preserve
```

```
        keep if Age >= 13
        tab Age, m
        tab yint11, m
restore

* ***********************************************************
* ***********************************************************

* ***********************************************************
* ***********************************************************
* declare survey design for dataset
* one-stage clustered design (with youth weight)
use     Data/YMM2/AdolescentsParents_complete, clear

svyset  cluster [pweight=YouthWeightC]

* generate subgroup identifying variable
gen     adolescents = (Age >= 13) if !missing(Age)
tab     adolescents
* correctly identified subpopulation. No one has missing Age!!
numlabel, add

/* subpopulation: adolescents with any perceived need
gen     ado_PN = (Age >= 13) if !missing(Age) & (needanyy == 1)
tab     ado_PN
* save  Data/YMM2/AdolescentsParents_complete, replace*/

* ***********************************************************
* ***********************************************************
* Missing Data: exclude cases with missing data
preserve
    keep if adolescents == 1
    misstable summarize sex IRSAD_dich Remoteness_dich family_type_dich par_education par_psychopathology ///
                        fadbi par_feelings totdiff_adoORpar externalising_adoORpar internalising_adoORpar
restore /* totally 2 missings in par_psychopathology and par_feelings */

preserve
    keep if adolescents == 1
    tab par_feelings par_psychopathology, m
restore /* not same person has missing  */

* percent missing
display (4/2314)*100

* generate subgroup adolescents complete (adoelscents_comp) to use for svy analyses
gen     adolescents_comp = (Age >= 13) if !missing(Age) & !missing(par_psychopathology) & !missing(par_feelings)
tab     adolescents_comp
```

```

* Reviewer 2: systematic differences in dropped and retained participants?
* parental psychopathology and parental feelings not included because that's where the missings are
preserve
    keep if adolescents == 1
    tab adolescents_comp, m
restore

recode  adolescents_comp (1=1) (.=0), pre(new_)

preserve
    keep if adolescents == 1
    foreach systematic in   sex IRSAD_dich Remoteness_dich family_type_dich par_education ///
                            fadbi totdiff_adoORpar {
                            logit `systematic' i.new_adolescents_comp, or
                            }
    mlogit  agree_need_outcome i.new_adolescents_comp
    regress Age i.new_adolescents_comp
restore

* cross-tab to see where problem lies
preserve
    keep if adolescents == 1
    foreach systematic in   IRSAD_dich fadbi {
                            tab `systematic' new_adolescents_comp, chi2 exact expected
                            }
restore
* zero cell frequencies. Expected cell frequencies <5 which violates assumptions of chi-square test. Fisher's exact test is an option.

* ************************************************************
* ************************************************************
* eTable 2 Sample characteristics
* total sample
foreach character in    sex IRSAD_Quintile IRSAD_dich Remoteness_dich family_type_dich par_education par_psychopathology ///
                        fadbi totdiff_adoORpar externalising_adoORpar internalising_adoORpar par_feelings {
                        svy, subpop(adolescents_comp): tab `character', percent cell se
}
svy, subpop(adolescents_comp): mean Age
estat sd

* results export to word
```

```
tabout      sex IRSAD_dich Remoteness_dich family_type_dich par_education par_psychopathology ///
            fadbi totdiff_adoORpar par_feelings using "Article/Tables_Figures/Results_Excel/Table1.txt", ///
            c(col se) f(3 3) clab(Row_% 95%_CI) svy npos(lab) percent ///
            replace ///
            style(txt) bt font(bold) cl1(2-6) /* this is complete rubbish */

* subsample excluding pairs that agreed on having no need
foreach character in    sex IRSAD_Quintile IRSAD_dich Remoteness_dich family_type_dich par_education par_psychopathology ///
                        fadbi totdiff_adoORpar externalising_adoORpar internalising_adoORpar par_feelings {
                        svy, subpop(if adolescents_comp == 1 & agree_need_outcome != 4): tab `character', percent cell se
}
svy, subpop(if adolescents_comp == 1 & agree_need_outcome != 4): mean Age
estat sd


* ****************************
* characteristics of those with any overall perceived need, adolescent report
foreach character in    sex IRSAD_Quintile IRSAD_dich Remoteness_dich family_type_dich par_education par_psychopathology ///
                            fadbi totdiff_adoORpar par_feelings {
                            svy, subpop(if adolescents_comp == 1 & needanyy == 1): ///
                            tab `character', percent cell se
    }
svy, subpop(if adolescents_comp == 1 & needanyy == 1): mean Age
estat sd

* ****************************
* characteristics of those with any overall perceived need, parent report
foreach character in    sex IRSAD_Quintile IRSAD_dich Remoteness_dich family_type_dich par_education par_psychopathology ///
                            fadbi totdiff_adoORpar par_feelings {
                            svy, subpop(if adolescents_comp == 1 & needany == 1): ///
                            tab `character', percent cell se
    }
svy, subpop(if adolescents_comp == 1 & needany == 1): mean Age
estat sd
```

```
* *****************************
* comparing adolescent and parent any overall perceived need
svy, subpop(adolescents_comp): tab needanyy needany, percent cell se

* ***********************************************************
* ***********************************************************
* Table 1 Overall Perceived Need and Types of Help Needed: group comparisons, agreement and kappa
* for reporting use design-based analysis (e.g. F statistic) because it accounts for survey design (weighting, clustering (& stratification))

* comparing perceived needs of adolescents and parents
* overall
svy, subpop(adolescents_comp): tab pneedby pneedb, percent cell pearson se
* effect size Cramer's V for svy:
local denom = e(r)-1
if e(c)< e(r){
local denom = e(c)-1
}
di "Cramer's V: " sqrt(e(cun_Pear)/(e(N)*`denom'))

* counselling
svy, subpop(adolescents_comp): tab pneed_counsy pneed_couns, percent cell pearson se
local denom = e(r)-1
if e(c)< e(r){
local denom = e(c)-1
}
di "Cramer's V: " sqrt(e(cun_Pear)/(e(N)*`denom'))

* medication
svy, subpop(adolescents_comp): tab pneed_medsy pneed_meds, percent cell pearson se
local denom = e(r)-1
if e(c)< e(r){
local denom = e(c)-1
}
di "Cramer's V: " sqrt(e(cun_Pear)/(e(N)*`denom'))

* information
svy, subpop(adolescents_comp): tab pneed_infoy pneed_info, percent cell pearson se
local denom = e(r)-1
if e(c)< e(r){
local denom = e(c)-1
}
di "Cramer's V: " sqrt(e(cun_Pear)/(e(N)*`denom'))

* skill training
```

```
svy, subpop(adolescents_comp): tab pneed_skillsy pneed_skills,
percent cell pearson se
local denom = e(r)-1
if e(c)< e(r){
local denom = e(c)-1
}
di "Cramer's V: " sqrt(e(cun_Pear)/(e(N)*`denom'))

* for right two columns in new Table 1 (by 13 March 2019) see at
the end of this file

* ****************************
* Table 2 kappa correlation - agreement (svy command does not
support kappa statistics)
* total agreement (four categories: unmet, partially met, fully
met, no need)
preserve
    keep if adolescents_comp == 1
    kap     pneedby pneedb, tab
    kap     pneed_counsy pneed_couns, tab
    kap     pneed_medsy pneed_meds, tab
    kap     pneed_infoy pneed_info, tab
    kap     pneed_skillsy pneed_skills, tab
restore

* agreement on any need (two categories: yes/no)
preserve
    keep if adolescents_comp == 1
    kap     needany needanyy, tab
restore

* ****************************
* comparing and kappa agreement of any need excluding no need
* overall need
preserve
    keep if adolescents_comp == 1 & agree_NO_overall == 0
    kap     pneedby pneedb, tab
restore

* need for counselling
preserve
    keep if adolescents_comp == 1 & agree_NO_counselling == 0
    kap     pneed_counsy pneed_couns, tab
restore

* need for medication
preserve
    keep if adolescents_comp == 1 & agree_NO_medication == 0
    kap     pneed_medsy pneed_meds, tab
restore

* need for information
```

```
preserve
    keep if adolescents_comp == 1 & agree_NO_info == 0
    kap     pneed_infoy pneed_info, tab
restore

* need for skill training
preserve
    keep if adolescents_comp == 1 & agree_NO_skill == 0
    kap     pneed_skillsy pneed_skills, tab
restore

* ***********************************************************
* ***********************************************************
* David: same as above but exclude the ones where both adolescents and parents report SDQs in normal range (agreement higher?)

* eTable 3
* comparing perceived needs of adolescents and parents
* overall
svy, subpop(if adolescents_comp == 1 & totdiff_adoORpar == 1): tab pneedby pneedb, percent cell pearson se
local denom = e(r)-1
if e(c)< e(r){
local denom = e(c)-1
}
di "Cramer's V: " sqrt(e(cun_Pear)/(e(N)*`denom'))

* counselling
svy, subpop(if adolescents_comp == 1 & totdiff_adoORpar == 1): tab pneed_counsy pneed_couns, percent cell pearson se
local denom = e(r)-1
if e(c)< e(r){
local denom = e(c)-1
}
di "Cramer's V: " sqrt(e(cun_Pear)/(e(N)*`denom'))

* medication
svy, subpop(if adolescents_comp == 1 & totdiff_adoORpar == 1): tab pneed_medsy pneed_meds, percent cell pearson se
local denom = e(r)-1
if e(c)< e(r){
local denom = e(c)-1
}
di "Cramer's V: " sqrt(e(cun_Pear)/(e(N)*`denom'))

* information
svy, subpop(if adolescents_comp == 1 & totdiff_adoORpar == 1): tab pneed_infoy pneed_info, percent cell pearson se
local denom = e(r)-1
if e(c)< e(r){
local denom = e(c)-1
```

```
}
di "Cramer's V: " sqrt(e(cun_Pear)/(e(N)*`denom'))

* skill training
svy, subpop(if adolescents_comp == 1 & totdiff_adoORpar == 1): tab pneed_skillsy pneed_skills, percent cell pearson se
local denom = e(r)-1
if e(c)< e(r){
local denom = e(c)-1
}
di "Cramer's V: " sqrt(e(cun_Pear)/(e(N)*`denom'))

* for right two columns in new eTable 3 (by 13 March 2019) see at the end of this file

* ****************************
* eTable 5
* kappa correlation - agreement
* total agreement (four categories: unmet, partially met, fully met, no need)
preserve
    keep if adolescents_comp == 1 & totdiff_adoORpar == 1
    kap     pneedby pneedb, tab
    kap     pneed_counsy pneed_couns, tab
    kap     pneed_medsy pneed_meds, tab
    kap     pneed_infoy pneed_info, tab
    kap     pneed_skillsy pneed_skills, tab
restore

* comparing and kappa agreement of any need excluding no need
* overall need
preserve
    keep if adolescents_comp == 1 & agree_NO_overall == 0 & totdiff_adoORpar == 1
    kap     pneedby pneedb, tab
restore

* need for counselling
preserve
    keep if adolescents_comp == 1 & agree_NO_counselling == 0 & totdiff_adoORpar == 1
    kap     pneed_counsy pneed_couns, tab
restore

* need for medication
preserve
    keep if adolescents_comp == 1 & agree_NO_medication == 0 & totdiff_adoORpar == 1
    kap     pneed_medsy pneed_meds, tab
restore

* need for information
```

```
preserve
    keep if adolescents_comp == 1 & agree_NO_info == 0 & totdiff_adoORpar == 1
    kap     pneed_infoy pneed_info, tab
restore

* need for skill training
preserve
    keep if adolescents_comp == 1 & agree_NO_skill == 0 & totdiff_adoORpar == 1
    kap     pneed_skillsy pneed_skills, tab
restore

* ***********************************************************
* ***********************************************************
* eTable 1 classification of patterns of agreement (N/n per category, without svy just unweighted numbers)
preserve
    keep if adolescents_comp == 1
    tab     agree_need_outcome, m
restore

* ***********************************************************
* ***********************************************************
* eTable 4 cell frequencies of agreement on overall perceived need (cross-tab)
preserve
    keep if adolescents_comp == 1
    tab     pneedby pneedb, m
    tab     pneed_counsy pneed_couns, m
    tab     pneed_medsy pneed_meds, m
    tab     pneed_infoy pneed_info, m
    tab     pneed_skillsy pneed_skills, m
restore

* ***********************************************************
* ***********************************************************
* there will be five options of outcome:
* 1 -   four groups of agreement: agree on fully met (reference category); agree on either partially met or unmet; ado need > parent; parent need > ado
* 2 -   three groups of agreement: agree on either fully met, partially met or unmet; ado need > parent; parent need > ado
* 3 -   two groups of agreement: agree on either fully met, partially met or unmet; disagreement
* 4 -   three groups of agreement: agree on fully met (reference category); agree on partially met or unmet; disagree
* 5 -   two groups of agreement: agree on fully met need (reference group, received what was needed); not received what was needed (incl.
*       agree on partially met or unmet, and disagreement)
* ***********************************************************
```

```
* ************************************************************

* ************************************************************
* Option 1 (decision on 18 December 2018: we go with the first option)

* *****************
* *****************
* four category outcome (agree_need_outcome) --> refernce category: agree on fully met need
* first: simple multinomial logistic regressions
* second: fully adjusted model

* Table 3 & eTable 6

* *****************
* first
* unadjusted: socio, family and clinical characteristics (ado or parent identified a problem)
* additionally according to reviewer 1 comment (split up probable disorder into internalising and externalising) (eTable 7)
foreach unadjusted in   i.sex i.IRSAD_dich i.Remoteness_dich ib1.family_type_dich i.par_education i.par_psychopathology ///
                        i.fadbi i.totdiff_adoORpar i.externalising_adoORpar i.internalising_adoORpar i.par_feelings {
    svy, subpop(if adolescents_comp == 1 & agree_need_outcome != 4): mlogit agree_need_outcome `unadjusted', base(0) rrr
    }

* *****************
* second
* fully adjusted (all socio, family and clinical variables in)
svy, subpop(if adolescents_comp == 1 & agree_need_outcome != 4): ///
mlogit agree_need_outcome   i.sex i.IRSAD_dich i.Remoteness_dich ib1.family_type_dich i.par_education i.par_psychopathology ///
                            i.fadbi i.totdiff_adoORpar i.par_feelings, base(0) rrr
mlogitgof, table
/* if mlogitgof is specified after this version of subgroup specification, it does not take subgroup into account
but computes it on basis of hole sample. Use estimates from subpop() command and mlogitgof from version bellow. SE slightly differs */

* use: other option to specify subpopulation
svy:    mlogit agree_need_outcome   i.sex i.IRSAD_dich i.Remoteness_dich ib1.family_type_dich i.par_education i.par_psychopathology ///
                                    i.fadbi i.totdiff_adoORpar i.par_feelings ///
        if adolescents_comp == 1 & agree_need_outcome != 4, base(
```

```
0) rrr
mlogitgof, table
/* indicates lack of model fit (p=0.009)
Clyde Schechter says this on https://www.statalist.org/forums/forum/general-stata-discussion/general/389818-goodness-of-fit-test-for-logistic-regression-on-survey-data:
"The problem with the Hosmer-Lemeshow test is precisely that it is a goodness of fit test,
and goodness of fit is really only minimally relevant for most practical purposes. The logistic model is almost always a mismatch to a
real-life data generating process. If you have a sufficiently large sample, that misfit will be detected, even if the model is doing a pretty
good job of matching predicted to observed probabilities. If you have a sufficiently small sample (it looks like you have around 80 observations),
when you divide them into deciles, as H-L does, you will have, optimally (if there are no ties), 8 in each group--so your power to detect even
substantive deviations between predicted and observed is going to be fairly low, and almost any model will pass muster on the p < 0.05 criterion."
--> use the -,table- to see whether option the model's predicted probabilities are close to observed probabilities and yes they look quite good.
*/

* discrete change: how do Predicted Probabilites change as IV changes (e.g. from 0 to 1)
* dydx after margins computes difference change between e.g. 0 and 1
margins /* reports predicted value of the DV for each observation. I.e. disagreement cat. are quite well predicted (ca. 40%) and agreement ones not (ca. 10%) */
margins totdiff_adoORpar sex family_type_dich fadbi /* predicted probabilities for significant variables on outcome holding rest constant */
margins, dydx(totdiff_adoORpar sex family_type_dich fadbi) /* average marginal effect if categories change holding rest constant */
marginsplot
coefplot, drop(_cons) xline(0) keep(*:)

* ****************
* second part deux: with reviewer's 1 variable
* fully adjusted (all socio, family and clinical variables in)
svy, subpop(if adolescents_comp == 1 & agree_need_outcome != 4): ///
mlogit agree_need_outcome   i.sex i.IRSAD_dich i.Remoteness_dich ib1.family_type_dich i.par_education i.par_psychopathology ///
```

```
                            i.fadbi i.externalising_adoORpar i.internalising_adoORpar i.par_feelings, base(0) rrr
* use: other option to specify subpopulation
svy:    mlogit agree_need_outcome   i.sex i.IRSAD_dich i.Remoteness_dich ib1.family_type_dich i.par_education i.par_psychopathology ///
                                    i.fadbi i.externalising_adoORpar i.internalising_adoORpar i.par_feelings ///
        if adolescents_comp == 1 & agree_need_outcome != 4, base(0) rrr
mlogitgof, table

* *****************
* sensitivity analysis 1: take variable 'parental knowledge of adolescents' feelings' from model and see what changes
svy, subpop(if adolescents_comp == 1 & agree_need_outcome != 4): ///
mlogit agree_need_outcome   i.sex i.IRSAD_dich i.Remoteness_dich ib1.family_type_dich i.par_education i.par_psychopathology ///
                            i.fadbi i.totdiff_adoORpar, base(0) rrr

svy:    mlogit agree_need_outcome   i.sex i.IRSAD_dich i.Remoteness_dich ib1.family_type_dich i.par_education i.par_psychopathology ///
                                    i.fadbi i.totdiff_adoORpar ///
        if adolescents_comp == 1 & agree_need_outcome != 4, base(0) rrr
mlogitgof, table
coefplot, drop(_cons) xline(0) keep(*:)

* *****************
* sensitivity analysis 2: same model subsample with a mental health problem (n=648) (eTable 8)
* first: unadjusted
foreach unadjusted in   i.sex i.IRSAD_dich i.Remoteness_dich ib1.family_type_dich i.par_education i.par_psychopathology ///
                        i.fadbi i.par_feelings {
    svy, subpop(if adolescents_comp == 1 & agree_need_outcome != 4 & totdiff_adoORpar == 1): mlogit agree_need_outcome `unadjusted', base(0) rrr
    }

* second: fully adjusted
svy, subpop(if adolescents_comp == 1 & agree_need_outcome != 4 & totdiff_adoORpar == 1): ///
mlogit agree_need_outcome   i.sex i.IRSAD_dich i.Remoteness_dich ib1.family_type_dich i.par_education i.par_psychopathology ///
                            i.fadbi i.par_feelings, base(0) rrr

svy:    mlogit agree_need_outcome   i.sex i.IRSAD_dich i.Remoteness_dich ib1.family_type_dich i.par_education ///
```

```
                                    i.par_psychopathology i.fadbi i.par_feelings ///
        if adolescents_comp == 1 & agree_need_outcome != 4 & totdiff_adoORpar == 1, base(0) rrr
mlogitgof, table

* ***********************************************************
/* Option 2

* *****************
* *****************
* three category outcome (agree_need_outcome_tri) --> reference category: agree on fully met, partially met or unmet need
* first: simple multinomial logistic regressions
* second: fully adjusted model
* third: only those categories that were sign in simple logistic regression

* *****************
* first
* unadjusted: socio, family and clinical characteristics (ado or parent identified a problem)
foreach unadjusted in   i.sex i.IRSAD_dich i.Remoteness_dich i.family_type_dich i.par_education i.par_psychopathology ///
                        i.fadbi i.totdiff_adoORpar i.par_feelings {
    svy, subpop(if adolescents_comp == 1 & agree_need_outcome_tri !=3): mlogit agree_need_outcome_tri `unadjusted', base(0) rrr
    }

* *****************
* second
svy:    mlogit agree_need_outcome_tri   i.sex i.IRSAD_dich i.Remoteness_dich i.family_type_dich i.par_education i.par_psychopathology ///
                                        i.fadbi i.totdiff_adoORpar i.par_feelings ///
        if adolescents_comp == 1 & agree_need_outcome_tri != 3, base(0) rrr
mlogitgof, table /* indicates ok model fit (p=0.145) */

* ***********************************************************
* Option 3

* *****************
* *****************
* binary outcome agree/disagree on perceived need (agree_need_outcome_dich)
* Logistic Regression
* pseudo R2 not reported because of cluster (pseudo R2 is computed using log likelihoods and they assume that cases are
```

```
independent of each other)

* ****************
* first
* unadjusted: socio, family and clinical characteristics (ado or parent identified a problem)
foreach unadjusted in   i.sex i.IRSAD_dich i.Remoteness_dich i.family_type_dich i.par_education i.par_psychopathology ///
                        i.fadbi i.totdiff_adoORpar i.par_feelings {
    svy, subpop(if adolescents_comp == 1 & agree_need_outcome_dich != 2): logistic agree_need_outcome_dich `unadjusted'
    }

* ****************
* second
* fully adjusted model based on earlier studies (all socio, family and clinical variables in)
svy: logistic   agree_need_outcome_dich i.sex i.IRSAD_dich i.Remoteness_dich i.family_type_dich i.par_education i.par_psychopathology ///
                i.fadbi i.totdiff_adoORpar i.par_feelings ///
                if adolescents_comp == 1 & agree_need_outcome_dich != 2
estat gof /* that works, model fit ok */
mlogitgof, table /* should be same as estat gof as logistic regression but not same result?! */
linktest /* if linktest not significant, there should not be a specification error.
* See: https://stats.idre.ucla.edu/stata/webbooks/logistic/chapter3/lesson-3-logistic-regression-diagnostics-2/ */

* predicted probabilities (only for sign. variables)
margins
margins totdiff_adoORpar sex par_education
margins, dydx(totdiff_adoORpar sex par_education) /* we expect that on average the probability of disagreement is 11% lower among those with a mh problem */
marginsplot, noci
margins, dydx(totdiff_adoORpar) at(sex=(0 1)) /* tests if margins for mh problems differ by sex (not really) */
margins, dydx(totdiff_adoORpar) at(par_education=(0 1)) /* tests if margins for mh problems differ by parental education (not really) */
coefplot, drop(_cons) xline(0) keep(*:)

* *****************
* *****************
* test assumptions for logistic regression
* 1: independence of observation --> ok
```

```
* 2: data must not show multicollinearity --> only problematic when main iv is highly correlated with a control variable (problem for interpretation)
preserve
    keep if agree_need_outcome_dich !=2
    regress agree_need_outcome_dich i.sex i.Remoteness_dich i.family_type_dich i.par_psychopathology ///
        i.par_education i.IRSAD_dich i.fadbi i.totdiff_adoORpar /* estat vif only works after regress not logit*/
    estat vif /* checks for multicollinearity betwenn dv --> no multicoll because VIF always only bit above 1.0 */
* 3: specification problem? see above linktest --> ok
* 4: linear relation between continuous iv and logit transformation of dv with boxtid (we don't have continous ivs...)
restore


* ***********************************************************
* Option 4

* *****************
* *****************
* three category outcome (agree_need_outcome_tri) --> reference category: agree on fully met, partially met or unmet need
* first: simple multinomial logistic regressions
* second: fully adjusted model
* third: only those categories that were sign in simple logistic regression

* *****************
* first
* unadjusted: socio, family and clinical characteristics (ado or parent identified a problem)
foreach unadjusted in   i.sex i.IRSAD_dich i.Remoteness_dich i.family_type_dich i.par_education i.par_psychopathology ///
                        i.fadbi i.totdiff_adoORpar i.par_feelings {
    svy, subpop(if adolescents_comp == 1 & agree_need_outcome_tri2 !=3): mlogit agree_need_outcome_tri2 `unadjusted', base(0) rrr
    }

* *****************
* second: fully adjusted (analysis for estimates)
svy, subpop(if adolescents_comp == 1 & agree_need_outcome_tri2 != 3): ///
mlogit agree_need_outcome_tri2  i.sex i.IRSAD_dich i.Remoteness_dich ib1.family_type_dich i.par_education i.par_psychopathology ///
                            i.fadbi i.totdiff_adoORpar i.par_feelings, base(0) rrr

```

```
* other specification of model for gof
svy:    mlogit agree_need_outcome_tri2  i.sex i.IRSAD_dich i.Remoteness_dich i.family_type_dich i.par_education i.par_psychopathology ///
                                        i.fadbi i.totdiff_adoORpar i.par_feelings ///
        if adolescents_comp == 1 & agree_need_outcome_tri2 != 3, base(0) rrr
mlogitgof, table /* indicates ok model fit (p=0.583) */

* ***********************************************************
* Option 5

* *****************
* *****************
* binary outcome agree/disagree on perceived need (agree_need_outcome_dich)
* Logistic Regression
* pseudo R2 not reported because of cluster (pseudo R2 is computed using log likelihoods and they assume that cases are independent of each other)

* *****************
* first
* unadjusted: socio, family and clinical characteristics (ado or parent identified a problem)
foreach unadjusted in   i.sex i.IRSAD_dich i.Remoteness_dich i.family_type_dich i.par_education i.par_psychopathology ///
                        i.fadbi i.totdiff_adoORpar i.par_feelings {
    svy, subpop(if adolescents_comp == 1 & agree_need_outcome_dich2 != 2): logistic agree_need_outcome_dich2 `unadjusted'
    }

* *****************
* second
* fully adjusted model (for estimates)
svy, subpop(if adolescents_comp == 1 & agree_need_outcome_dich2 != 2): logistic agree_need_outcome_dich2 ///
                i.sex i.IRSAD_dich i.Remoteness_dich i.family_type_dich i.par_education i.par_psychopathology ///
                i.fadbi i.totdiff_adoORpar i.par_feelings

* for gof
svy: logistic   agree_need_outcome_dich2 i.sex i.IRSAD_dich i.Remoteness_dich i.family_type_dich i.par_education i.par_psychopathology ///
                i.fadbi i.totdiff_adoORpar i.par_feelings ///
                if adolescents_comp == 1 & agree_need_outcome_dich2 != 2
estat gof */
```

```
* ***********************************************************
* ***********************************************************
* eTable 9
* Any barriers to care separately for adolescents and parents (see also plot in R, Figure)
preserve
    keep if adolescents_comp == 1
    tab agree_need_barrier, m
restore

* total subsample where either adolescent or parent identified fully or partially unmet need
foreach barrier0 in AnyAttitAdo AnyStructAdo AnyAttitPar AnyStructPar {
            svy, subpop(adolescents_comp):  tab `barrier0', percent cell se
    }

* adolescents and parents have fully or partially unmet need (agree_need_barrier == 1)
foreach barrier1 in AnyAttitAdo AnyStructAdo AnyAttitPar AnyStructPar {
            svy, subpop(if adolescents_comp == 1 & agree_need_barrier == 1): tab `barrier1', percent cell se
    }

    * cross-tab for this sub-sample (ado vs. parent)
    svy, subpop(if adolescents_comp == 1 & agree_need_barrier == 1): tab AnyAttitAdo AnyAttitPar, percent cell pearson se
    local denom = e(r)-1
    if e(c)< e(r){
    local denom = e(c)-1
    }
    di "Cramer's V: " sqrt(e(cun_Pear)/(e(N)*`denom'))



* adolescents only have fully or partially unmet need (agree_need_barrier == 2)
foreach barrier2 in AnyAttitAdo AnyStructAdo {
            svy, subpop(if adolescents_comp == 1 & agree_need_barrier == 2): tab `barrier2', percent cell se
    }

* parents only have fully or partially unmet need (agree_need_barrier == 3)
foreach barrier3 in AnyAttitPar AnyStructPar {
            svy, subpop(if adolescents_comp == 1 & agree_need_barrier == 3): tab `barrier3', percent cell se
    }
```

```
* ***********************
* eTable 9 continued
* Single barriers to care

* adolescent identified unmet or partially met need
foreach barrier1 in     ado_stigma ado_selfrel ado_unsneed ado_unswhere ado_bettself ado_getserv ado_cost ado_appoint ado_school {
            svy, subpop(adolescents_comp):  tab `barrier1', percent cell se
    }

* parent identified unmet or partially met need
foreach barrier1 in par_stigma par_selfrel par_unsneed par_unswhere par_bettself par_refuse par_getserv par_cost par_appoint {
            svy, subpop(adolescents_comp):  tab `barrier1', percent cell se
    }

* adolescents and parents have fully or partially unmet need (agree_need_barrier == 1)
foreach barrier1 in par_stigma par_selfrel par_unsneed par_unswhere par_bettself par_refuse par_getserv par_cost par_appoint ///
                        ado_stigma ado_selfrel ado_unsneed ado_unswhere ado_bettself ado_getserv ado_cost ado_appoint ado_school {
            svy, subpop(if adolescents_comp == 1 & agree_need_barrier == 1): tab `barrier1', percent cell se
    }

* adolescents only have fully or partially unmet need (agree_need_barrier == 2)
foreach barrier2 in ado_stigma ado_selfrel ado_unsneed ado_unswhere ado_bettself ado_getserv ado_cost ado_appoint ado_school {
            svy, subpop(if adolescents_comp == 1 & agree_need_barrier == 2): tab `barrier2', percent cell se
    }

* parents only have fully or partially unmet need (agree_need_barrier == 3)
foreach barrier3 in par_stigma par_selfrel par_unsneed par_unswhere par_bettself par_refuse par_getserv par_cost par_appoint {
            svy, subpop(if adolescents_comp == 1 & agree_need_barrier == 3): tab `barrier3', percent cell se
    }

* ***********************
* adolescent-parent agreement on barriers among the subsample
```

```
where both report an either unmet or partially met need
preserve
    keep if adolescents_comp == 1 & agree_need_barrier == 1
    kap AnyAttitAdo AnyAttitPar, tab
    kap AnyStructAdo AnyStructPar, tab
restore

* ***********************************************************
* ***********************************************************
* comment Michael regarding that 1/3 of either ados or parents report a perceived need for care and this likely relating to different individuals
* how frequent is any need, partially met need etc. if adolescents or parents report it?
* Table 1 addition

* ************************
* any type of help
gen     pneed_adoORpar = .
replace pneed_adoORpar = 0 if agree_need_outcome == 4
replace pneed_adoORpar = 1 if agree_need_outcome == 0 | agree_need_outcome == 1 | agree_need_outcome == 2 | agree_need_outcome == 3
la de   PNadoORpar 0 "ado and parent no perceived need" 1 "ado or parent any perceived need"
la val  pneed_adoORpar PNadoORpar
la var  pneed_adoORpar "Either adolescent or parent perceive a need for any type of care"
tab     pneed_adoORpar, m
* missings = ados that did not respond to self-report
tab     pneed_adoORpar

* % of total population where either ado or parent reported a need for any type of care
svy, subpop(adolescents_comp): tab pneed_adoORpar, percent cell se

* how many % of adolescents or parents have fully met, partially met, unmet need among those where either one of them has a PN for any type of care
svy, subpop(if adolescents_comp == 1 & pneed_adoORpar == 1): tab pneedby pneedb, percent cell pearson se
* Cramer's V:
local denom = e(r)-1
if e(c)< e(r){
local denom = e(c)-1
}
di "Cramer's V: " sqrt(e(cun_Pear)/(e(N)*`denom'))

* ************************
* counselling
gen     pneedcouns_adoORoar = .
replace pneedcouns_adoORoar = 0 if  pneed_couns == 4 &
```

```
pneed_counsy == 4
replace pneedcouns_adoORoar = 1 if  pneed_couns == 1 & pneed_counsy == 1 | ///
                                    pneed_couns == 2 & pneed_counsy == 2 | pneed_couns == 3 & pneed_counsy == 3 | ///
                                    pneed_counsy == 3 & pneed_couns == 2 | pneed_counsy == 3 & pneed_couns == 1 | pneed_counsy == 3 & pneed_couns == 4 | ///
                                    pneed_counsy == 2 & pneed_couns == 1 | pneed_counsy == 2 & pneed_couns == 4 | ///
                                    pneed_counsy == 1 & pneed_couns == 4 | ///
                                    pneed_couns == 3 & pneed_counsy == 2 | pneed_couns == 3 & pneed_counsy == 1 | pneed_couns == 3 & pneed_counsy == 4 | ///
                                    pneed_couns == 2 & pneed_counsy == 1 | pneed_couns == 2 & pneed_counsy == 4 | ///
                                    pneed_couns == 1 & pneed_counsy == 4
la val  pneedcouns_adoORoar PNadoORpar
la var  pneedcouns_adoORoar "Either adolescent or parent perceive a need for counselling"
tab     pneedcouns_adoORoar, m

* % of total population where either ado or parent reported a need for counselling
svy, subpop(adolescents_comp): tab pneedcouns_adoORoar, percent cell se

* how many % of adolescents or parents have fully met, partially met, unmet need among those where either one of them has a PN for counselling
svy, subpop(if adolescents_comp == 1 & pneedcouns_adoORoar == 1): tab pneed_counsy pneed_couns, percent cell pearson se
local denom = e(r)-1
if e(c)< e(r){
local denom = e(c)-1
}
di "Cramer's V: " sqrt(e(cun_Pear)/(e(N)*`denom'))

* ************************
* medication
gen     pneedmeds_adoORoar = .
replace pneedmeds_adoORoar = 0 if   pneed_meds == 4 & pneed_medsy == 4
replace pneedmeds_adoORoar = 1 if   pneed_meds == 1 & pneed_medsy == 1 | ///
                                    pneed_meds == 2 & pneed_medsy == 2 | pneed_meds == 3 & pneed_medsy == 3 | ///
                                    pneed_medsy == 3 & pneed_meds == 2 | pneed_medsy == 3 & pneed_meds == 1 | pneed_medsy == 3 & pneed_meds == 4 | ///
```

```
                                    pneed_medsy == 2 & pneed_meds == 1 | pneed_medsy == 2 & pneed_meds == 4 | ///
                                    pneed_medsy == 1 & pneed_meds == 4 | ///
                                    pneed_meds == 3 & pneed_medsy == 2 | pneed_meds == 3 & pneed_medsy == 1 | pneed_meds == 3 & pneed_medsy == 4 | ///
                                    pneed_meds == 2 & pneed_medsy == 1 | pneed_meds == 2 & pneed_medsy == 4 | ///
                                    pneed_meds == 1 & pneed_medsy == 4
la val  pneedmeds_adoORoar PNadoORpar
la var  pneedmeds_adoORoar "Either adolescent or parent perceive a need for medication"
tab     pneedmeds_adoORoar, m

* % of total population where either ado or parent reported a need for medication
svy, subpop(adolescents_comp): tab pneedmeds_adoORoar, percent cell se

* how many % of adolescents or parents have fully met, partially met, unmet need among those where either one of them has a PN for medication
svy, subpop(if adolescents_comp == 1 & pneedmeds_adoORoar == 1): tab pneed_medsy pneed_meds, percent cell pearson se
local denom = e(r)-1
if e(c)< e(r){
local denom = e(c)-1
}
di "Cramer's V: " sqrt(e(cun_Pear)/(e(N)*`denom'))

* ************************
* information
gen     pneedinfo_adoORoar = .
replace pneedinfo_adoORoar = 0 if   pneed_info == 4 & pneed_infoy == 4
replace pneedinfo_adoORoar = 1 if   pneed_info == 1 & pneed_infoy == 1 | ///
                                    pneed_info == 2 & pneed_infoy == 2 | pneed_info == 3 & pneed_infoy == 3 | ///
                                    pneed_infoy == 3 & pneed_info == 2 | pneed_infoy == 3 & pneed_info == 1 | pneed_infoy == 3 & pneed_info == 4 | ///
                                    pneed_infoy == 2 & pneed_info == 1 | pneed_infoy == 2 & pneed_info == 4 | ///
                                    pneed_infoy == 1 & pneed_info == 4 | ///
                                    pneed_info == 3 & pneed_infoy == 2 | pneed_info == 3 & pneed_infoy == 1 | pneed_info == 3 & pneed_infoy == 4 | ///
                                    pneed_info == 2 & pneed_infoy
```

```
 == 1 | pneed_info == 2 & pneed_infoy == 4 | ///
                                    pneed_info == 1 & pneed_infoy == 4
la val  pneedinfo_adoORoar PNadoORpar
la var  pneedinfo_adoORoar "Either adolescent or parent perceive a need for information"
tab     pneedinfo_adoORoar, m

* % of total population where either ado or parent reported a need for information
svy, subpop(adolescents_comp): tab pneedinfo_adoORoar, percent cell se

* how many % of adolescents or parents have fully met, partially met, unmet need among those where either one of them has a PN for information
svy, subpop(if adolescents_comp == 1 & pneedinfo_adoORoar == 1): tab pneed_infoy pneed_info, percent cell pearson se
local denom = e(r)-1
if e(c)< e(r){
local denom = e(c)-1
}
di "Cramer's V: " sqrt(e(cun_Pear)/(e(N)*`denom'))

* ************************
* skill training
gen     pneedskills_adoORoar = .
replace pneedskills_adoORoar = 0 if pneed_skills == 4 & pneed_skillsy == 4
replace pneedskills_adoORoar = 1 if pneed_skills == 1 & pneed_skillsy == 1 | ///
                                    pneed_skills == 2 & pneed_skillsy == 2 | pneed_skills == 3 & pneed_skillsy == 3 | ///
                                    pneed_skillsy == 3 & pneed_skills == 2 | pneed_skillsy == 3 & pneed_skills == 1 | pneed_skillsy == 3 & pneed_skills == 4 | ///
                                    pneed_skillsy == 2 & pneed_skills == 1 | pneed_skillsy == 2 & pneed_skills == 4 | ///
                                    pneed_skillsy == 1 & pneed_skills == 4 | ///
                                    pneed_skills == 3 & pneed_skillsy == 2 | pneed_skills == 3 & pneed_skillsy == 1 | pneed_skills == 3 & pneed_skillsy == 4 | ///
                                    pneed_skills == 2 & pneed_skillsy == 1 | pneed_skills == 2 & pneed_skillsy == 4 | ///
                                    pneed_skills == 1 & pneed_skillsy == 4
la val  pneedskills_adoORoar PNadoORpar
la var  pneedskills_adoORoar "Either adolescent or parent perceive a need for skill training"
tab     pneedskills_adoORoar, m
```

```
* % of total population where either ado or parent reported a need for skill training
svy, subpop(adolescents_comp): tab pneedskills_adoORoar, percent cell se

* how many % of adolescents or parents have fully met, partially met, unmet need among those where either one of them has a PN for skill training
svy, subpop(if adolescents_comp == 1 & pneedskills_adoORoar == 1 ): tab pneed_skillsy pneed_skills, percent cell pearson se
local denom = e(r)-1
if e(c)< e(r){
local denom = e(c)-1
}
di "Cramer's V: " sqrt(e(cun_Pear)/(e(N)*`denom'))

* ************************
* ************************
* eTable 3 addition
* comparing perceived needs of adolescents and parents
* overall
svy, subpop(if adolescents_comp == 1 & totdiff_adoORpar == 1 & pneed_adoORpar == 1): tab pneedby pneedb, percent cell pearson se
local denom = e(r)-1
if e(c)< e(r){
local denom = e(c)-1
}
di "Cramer's V: " sqrt(e(cun_Pear)/(e(N)*`denom'))

* counselling
svy, subpop(if adolescents_comp == 1 & totdiff_adoORpar == 1 & pneedcouns_adoORoar == 1): tab pneed_counsy pneed_couns, percent cell pearson se
local denom = e(r)-1
if e(c)< e(r){
local denom = e(c)-1
}
di "Cramer's V: " sqrt(e(cun_Pear)/(e(N)*`denom'))

* medication
svy, subpop(if adolescents_comp == 1 & totdiff_adoORpar == 1 & pneedmeds_adoORoar == 1): tab pneed_medsy pneed_meds, percent cell pearson se
local denom = e(r)-1
if e(c)< e(r){
local denom = e(c)-1
}
di "Cramer's V: " sqrt(e(cun_Pear)/(e(N)*`denom'))

* information
svy, subpop(if adolescents_comp == 1 & totdiff_adoORpar == 1 & pneedinfo_adoORoar == 1): tab pneed_infoy pneed_info, percent
```

```
cell pearson se
local denom = e(r)-1
if e(c)< e(r){
local denom = e(c)-1
}
di "Cramer's V: " sqrt(e(cun_Pear)/(e(N)*`denom'))

* skill training
svy, subpop(if adolescents_comp == 1 & totdiff_adoORpar == 1 & pneedskills_adoORoar == 1): tab pneed_skillsy pneed_skills, percent cell pearson se
local denom = e(r)-1
if e(c)< e(r){
local denom = e(c)-1
}
di "Cramer's V: " sqrt(e(cun_Pear)/(e(N)*`denom'))

* % of total sample with probable disorder where either ado aor parent reported a need for any and each type of help
foreach oftotal in      pneed_adoORpar pneedcouns_adoORoar pneedmeds_adoORoar pneedinfo_adoORoar pneedskills_adoORoar {
                        svy, subpop(if adolescents_comp == 1 & totdiff_adoORpar == 1): tab `oftotal', percent cell se
}

* ***********************************************************
* ***********************************************************
* Reviewer 2 Cronbach's alpha for SDQ (total difficulties) (from scale scores)
use     Data/YMM2/cleaned_merged/YouthParents_merged, clear

* parents
preserve
    keep if Age >= 13
    alpha   ppeer phyper pemotion pconduct, std item
restore

* adolescents
alpha   Ypeer Yhyper Yemotion Yconduct, std item

* Cronbach's alpha for SDQ (probable internalising or externalising disorder)
* parents internalising
preserve
    keep if Age >= 13
    alpha   ppeer pemotion, std item
restore

* parents externalising
preserve
    keep if Age >= 13
    alpha   phyper pconduct, std item
```

```
restore

* adolescents internalising
alpha   Ypeer Yemotion, std item

* adolescents externalising
alpha   Yhyper Yconduct, std item

* ***********
* Cronbach's alpha for K10 (parents) and for McMaster Family
Functioning Scale
use     Data/YMM2/OriginalData/parents

preserve
    keep if Age >= 13
    alpha   PFI1-PFI10, std item
    alpha   PFI22B-PFI22L, std item
restore
```

Survey Kappa

Specify comlex survey design and subpopulation

Stata's svy command does not support kappa statistics. R has this option in the package 'survey'.

```
# one-stage cluster sample (we don't have variables for multistage sampling to include: https://www.sta
YMM_survey <- svydesign(id = ~cluster,
                        probs = NULL,
                        strat = NULL,
                        weight = ~YouthWeightC,
                        data = YMM_PN,
                        nest = FALSE)

# restrict survey design to subpopulation
YMM_surv_ado <- subset(YMM_survey, Age>=13)
```

Survey estimates

We compared the survey estimates of Stata (command svy: tab) with those of R (command svytable).

```
# Note that the `echo = FALSE` parameter in code chunk above would prevent printing of the R code that 
(tbl_PN0 <- svytable(~pneedb+pneedby, YMM_surv_ado)) # returns population count, not proportions
```

```
##                       pneedby
## pneedb                 Fully met need Partially met need Unmet need
##   Fully met need            76638.066          33545.025  16836.717
##   Partially met need        37166.779          39026.063   7707.256
##   Unmet need                12917.666           8916.073  14117.845
##   No need                  113796.348          48683.640  59421.803
##                       pneedby
## pneedb                     No need
##   Fully met need         78417.113
##   Partially met need     44260.377
##   Unmet need             72181.154
##   No need               759581.630
```

```
plot(tbl_PN0) # funny plot
```

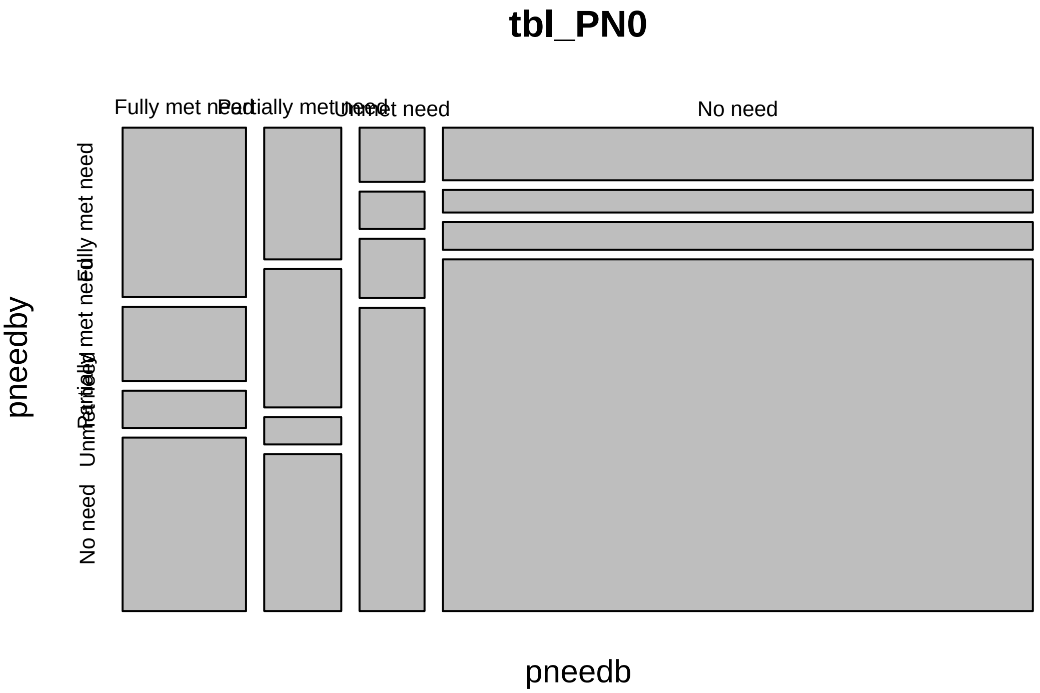


```r
svychisq(~pneedb+pneedby, YMM_surv_ado) # chi-square with F statistic: same results as in stata
```

```
##
##  Pearson's X^2: Rao & Scott adjustment
##
## data:  svychisq(~pneedb + pneedby, YMM_surv_ado)
## F = 37.801, ndf = 8.8023, ddf = 4709.2321, p-value < 2.2e-16
```

```r
prop <- svymean(~pneedb, YMM_surv_ado)  # gives proportion for each category of variable pneedb
prop
```

```
##                               mean     SE
## pneedbFully met need     0.144347 0.0081
## pneedbPartially met need 0.090050 0.0064
## pneedbUnmet need         0.075978 0.0067
## pneedbNo need            0.689625 0.0111
```

Survey kappa

How does survey kappa estimated in R compare with kappa estimated in stat?

```r
kapOvPN <-
  svykappa(~pneedb+pneedby, YMM_surv_ado)
kapOvPN # leads to the same estimate as just using 'normal' kappa. SE 0.02 (rounded) bit different.
```

```
##         nlcon     SE
## kappa 0.24928 0.0178
```

```r
# kappa for overall perceived need (PN) excluding those with agreement on no need ####
YMM_surv_ado_excl <- subset(YMM_surv_ado, agree_NO_overall == "no")
svytable(~agree_NO_overall, YMM_surv_ado_excl)
```

```
## agree_NO_overall
##       no      yes
```

```
## 663631.9      0.0
```

```
svytable(~pneedb+pneedby, YMM_surv_ado_excl)
```

```
##                       pneedby
## pneedb                 Fully met need Partially met need Unmet need
##   Fully met need            76638.066          33545.025  16836.717
##   Partially met need        37166.779          39026.063   7707.256
##   Unmet need                12917.666           8916.073  14117.845
##   No need                  113796.348          48683.640  59421.803
##                       pneedby
## pneedb                     No need
##   Fully met need      78417.113
##   Partially met need  44260.377
##   Unmet need          72181.154
##   No need                 0.000
```

```
kapOvPNex <-
  svykappa(~pneedb+pneedby, YMM_surv_ado_excl)
kapOvPNex # leads to same estimate and SE as using 'normal' kappa.
```

```
##          nlcon     SE
## kappa -0.10551 0.0192
```

Survey kappa in R leads to same estimates and SE as kappa estimated in stata without survey prefix.

R script for graphs

Perceived Need

Plot for overall perceived need for care (any type of care) and for perceived need for four different types of care (information, medication, counselling, skill-training)

Same plot as above but only including subsample of adolescents with a mental health problem.

Agreement on overall perceived need

Circos https://jokergoo.github.io/circlize_book/book/the-chorddiagram-function.html to plot agreement and disagreement of adolescents and parents on overall perceived need (for any type of care).

Subsetting and preparation

```
library(circlize)
library(janitor) # used to clean names

Overall <- tab(YMM_pn, pneedb, pneedby)
```

```
##
##              pneedb              pneedby      Freq.  Percent      Cum.
##
##      Fully met need       Fully met need        131     5.66      5.66
##      Fully met need   Partially met need         59     2.55      8.21
##      Fully met need           Unmet need         25     1.08      9.29
##      Fully met need              No need        125     5.40     14.69
## -------------------- -------------------- ----------------------------
## Partially met need       Fully met need         65     2.81     17.50
## Partially met need   Partially met need         70     3.03     20.53
## Partially met need           Unmet need         13     0.56     21.09
## Partially met need              No need         63     2.72     23.81
## -------------------- -------------------- ----------------------------
##          Unmet need       Fully met need         25     1.08     24.89
##          Unmet need   Partially met need         17     0.73     25.63
##          Unmet need           Unmet need         23     0.99     26.62
##          Unmet need              No need        113     4.88     31.50
## -------------------- -------------------- ----------------------------
##             No need       Fully met need        197     8.51     40.02
##             No need   Partially met need         84     3.63     43.65
##             No need           Unmet need        112     4.84     48.49
##             No need              No need       1192    51.51    100.00
```

```
Overall <- as.data.frame(Overall[, c(1:3)])
Overall <- plyr::rename(Overall, c("pneedb" = "Overall Need Parents",
                              "pneedby" = "Overall Need Adolescents",
                              "Freq." = "Frequency"))

Overall_agg <- Overall %>%
  clean_names()
rm(Overall)
```

Circos Plot

```r
# total sample ####

# Values for adults & kids cannot be the same, otherwise we get rubbish :/
Overall_agg$overall_need_parents <- paste("P:", Overall_agg$overall_need_parents)
Overall_agg$overall_need_adolescents <- paste("A:", Overall_agg$overall_need_adolescents)

circos.clear()
chordDiagram(Overall_agg)
```

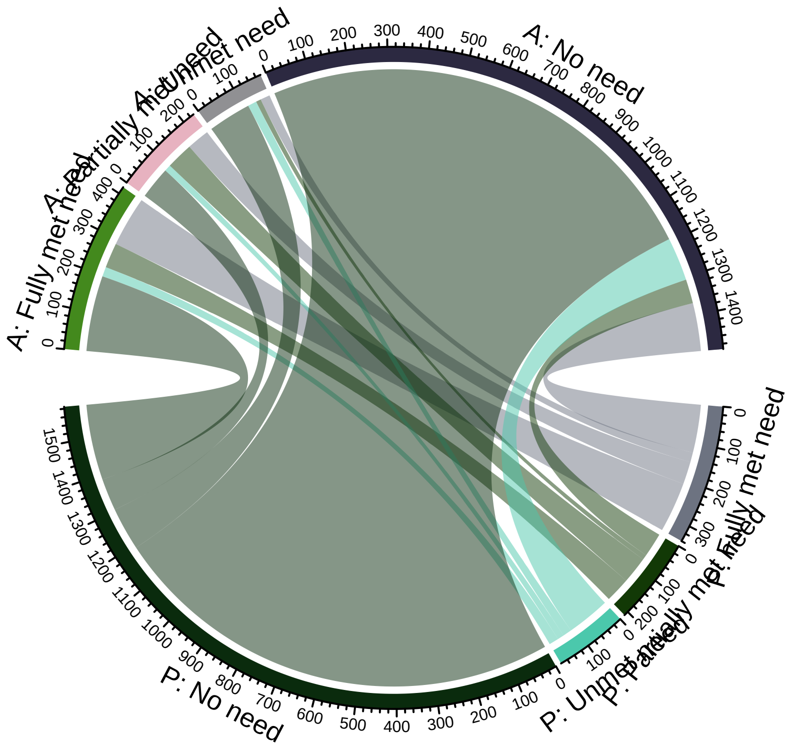


```r
# there is an option to specify order...
circos.clear() # always use this to reset to default graphical settings
chordDiagram(Overall_agg,
             order = c("P: Fully met need", "P: Partially met need", "P: Unmet need", "P: No need",
                       "A: Fully met need", "A: Partially met need", "A: Unmet need", "A: No need"))
```

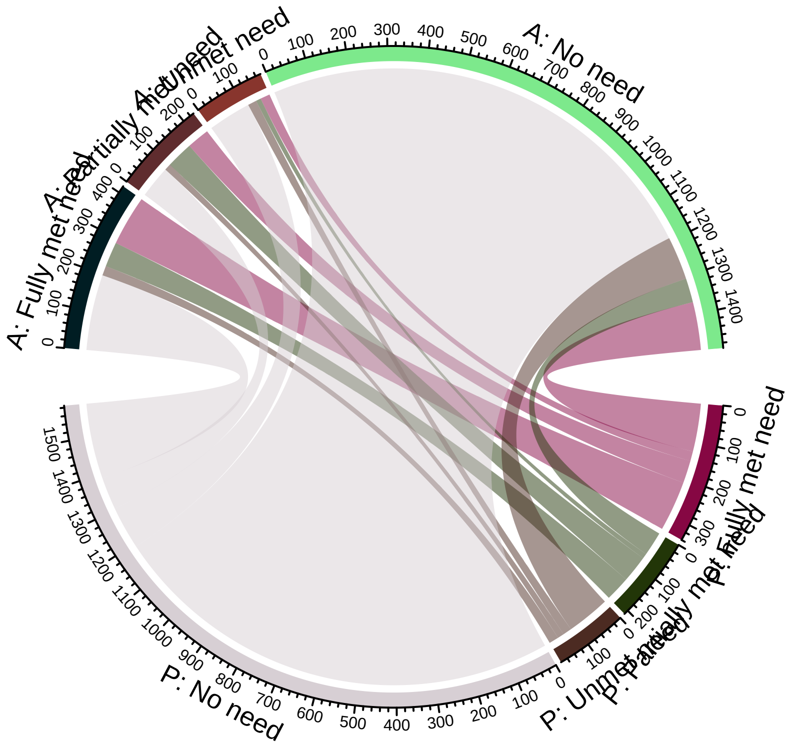


```r
# ... and to specify grid colours: same categories have same colours
grid.col = c("P: Fully met need" = "#7b3294", "P: Partially met need" = "#c2a5cf",
             "P: Unmet need" = "#80cdc1", "P: No need" = "#018571",
             "A: Fully met need" = "#7b3294", "A: Partially met need" = "#c2a5cf",
             "A: Unmet need" = "#80cdc1", "A: No need" = "#018571")

# ... link borders: add border/frame to agreement on each category
border_df = data.frame(c("P: Fully met need", "P: Partially met need", "P: Unmet need", "P: No need"),
                       c("A: Fully met need", "A: Partially met need", "A: Unmet need", "A: No need"),
                       c(1, 1, 1, 1))
# ... thickness of borders
lwd_df = data.frame(c("P: Fully met need", "P: Partially met need", "P: Unmet need", "P: No need"),
                    c("A: Fully met need", "A: Partially met need", "A: Unmet need", "A: No need"),
                    c(2, 2, 2, 2))

circos.clear()
chordOverall <- chordDiagram(Overall_agg,
                order = c("P: Fully met need", "P: Partially met need", "P: Unmet need", "P: No need",
                          "A: Fully met need", "A: Partially met need", "A: Unmet need", "A: No need"),
                grid.col = grid.col,
                transparency = 0.2,
                link.border = border_df,
                link.lwd = lwd_df)
title("Overall Perceived Need")
```

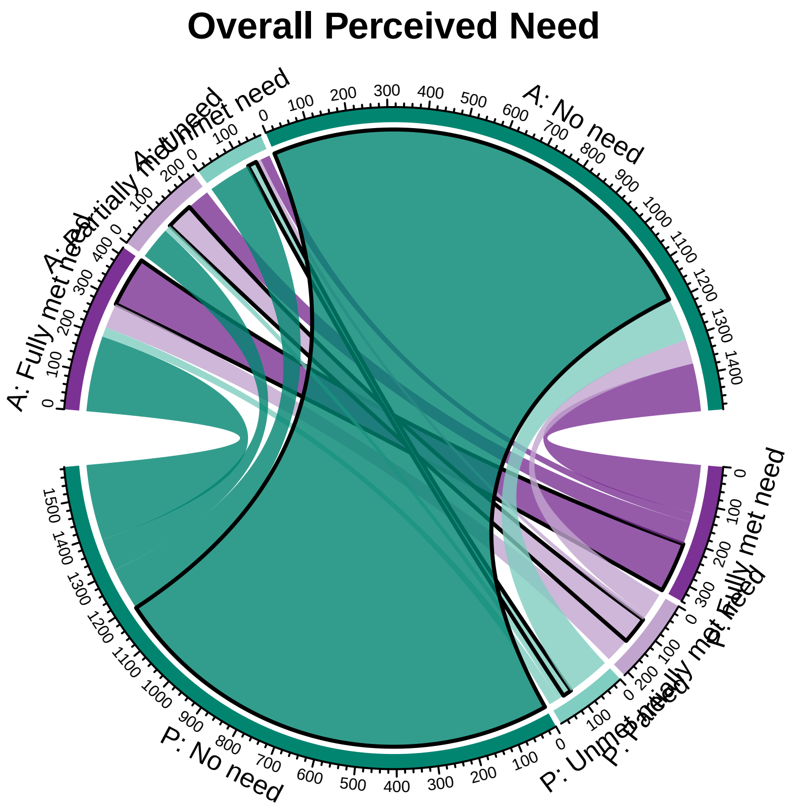

Barriers to care

Next, we will have a look at the distribution of adolescents and parents barriers to care (first without then second with survey weighted including confidence intervals).

```
# work with very long data and facet_wrap ####
YMM_barrier <- select(YMM_ado, householdID, par_selfrel:ado_school)
names(YMM_barrier)
```

```
##  [1] "householdID"  "par_selfrel"  "par_unsneed"  "par_unswhere"
##  [5] "par_bettself" "par_getserv"  "par_cost"     "par_appoint"
##  [9] "par_stigma"   "par_refuse"   "ado_selfrel"  "ado_unsneed"
## [13] "ado_unswhere" "ado_bettself" "ado_getserv"  "ado_cost"
## [17] "ado_appoint"  "ado_stigma"   "ado_school"
```

```
YMM_barrier[, c(2:19)] <-
  lapply(YMM_barrier[, c(2:19)], factor,
         labels = c("no", "yes"))
table(YMM_barrier$par_selfrel)
```

```
##
##  no yes
## 284 119
```

```
#### first: long data sets for adolescents and parents separately ####
# parents
YMM_barrier_parents <- select(YMM_barrier, par_selfrel:par_refuse)
YMM_barrier_parents$par_school <- NA
YMM_barrier_parents <- plyr::rename(YMM_barrier_parents, c("par_selfrel" = "self-reliance (A)",
```

```
                                                    "par_unsneed" = "unsure if help needed (A)",
                                                    "par_unswhere" = "unsure where to get help (A)",
                                                    "par_bettself" = "problem will get better by itsel
                                                    "par_getserv" = "problem finding service that coul
                                                    "par_cost" = "couldn't afford it (S)",
                                                    "par_appoint" = "couldn't get appointment (S)",
                                                    "par_stigma" = "stigma (A)",
                                                    "par_refuse" = "child refused",
                                                    "par_school" = "asked for help at school but didn'

YMM_barrier_long_parents <- YMM_barrier_parents %>%
  gather("self-reliance (A)", "unsure if help needed (A)",
         "unsure where to get help (A)", "problem will get better by itself (A)",
         "problem finding service that could help (S)", "couldn't afford it (S)",
         "couldn't get appointment (S)", "stigma (A)",
         "child refused", "asked for help at school but didn't get it (S)",
         key = "question", value = "noyes")
```

```
## Warning: attributes are not identical across measure variables;
## they will be dropped
```

```
YMM_barrier_long_parents$source <- "parents"

# adolescents
YMM_barrier_adolescents <- select(YMM_barrier, ado_selfrel:ado_school)
YMM_barrier_adolescents$ado_refuse <- NA
YMM_barrier_adolescents <- plyr::rename(YMM_barrier_adolescents, c("ado_selfrel" = "self-reliance (A)",
                                                        "ado_unsneed" = "unsure if help needed (A)
                                                        "ado_unswhere" = "unsure where to get help
                                                        "ado_bettself" = "problem will get better
                                                        "ado_getserv" = "problem finding service t
                                                        "ado_cost" = "couldn't afford it (S)",
                                                        "ado_appoint" = "couldn't get appointment
                                                        "ado_stigma" = "stigma (A)",
                                                        "ado_refuse" = "child refused",
                                                        "ado_school" = "asked for help at school b

YMM_barrier_long_adolescents <- YMM_barrier_adolescents %>%
  gather("self-reliance (A)", "unsure if help needed (A)",
         "unsure where to get help (A)", "problem will get better by itself (A)",
         "problem finding service that could help (S)", "couldn't afford it (S)",
         "couldn't get appointment (S)", "stigma (A)",
         "child refused", "asked for help at school but didn't get it (S)",
         key = "question", value = "noyes")
```

```
## Warning: attributes are not identical across measure variables;
## they will be dropped
```

```
YMM_barrier_long_adolescents$source <- "adolescents"

#### second: merge two long data sets into one very long ####
YMM_barrier_long <- rbind(YMM_barrier_long_parents, YMM_barrier_long_adolescents)

#### third: plot with facet_wrap ####
ggplot(na.omit(YMM_barrier_long), mapping = aes(x= source, fill = noyes)) +
```

```
  geom_bar(position = "fill") +
  facet_wrap(~question, nrow = 5, ncol = 2) + coord_flip() + xlab("") + ylab("proportion") +
  ggtitle("Barriers to care of adolescents and parents") +
  scale_fill_manual("", values = c("#c2a5cf", "#7b3294")) +
  theme(panel.grid.major.x = element_line(colour = "grey"), panel.background = element_blank(),
        axis.line = element_line(colour = "grey"))
```

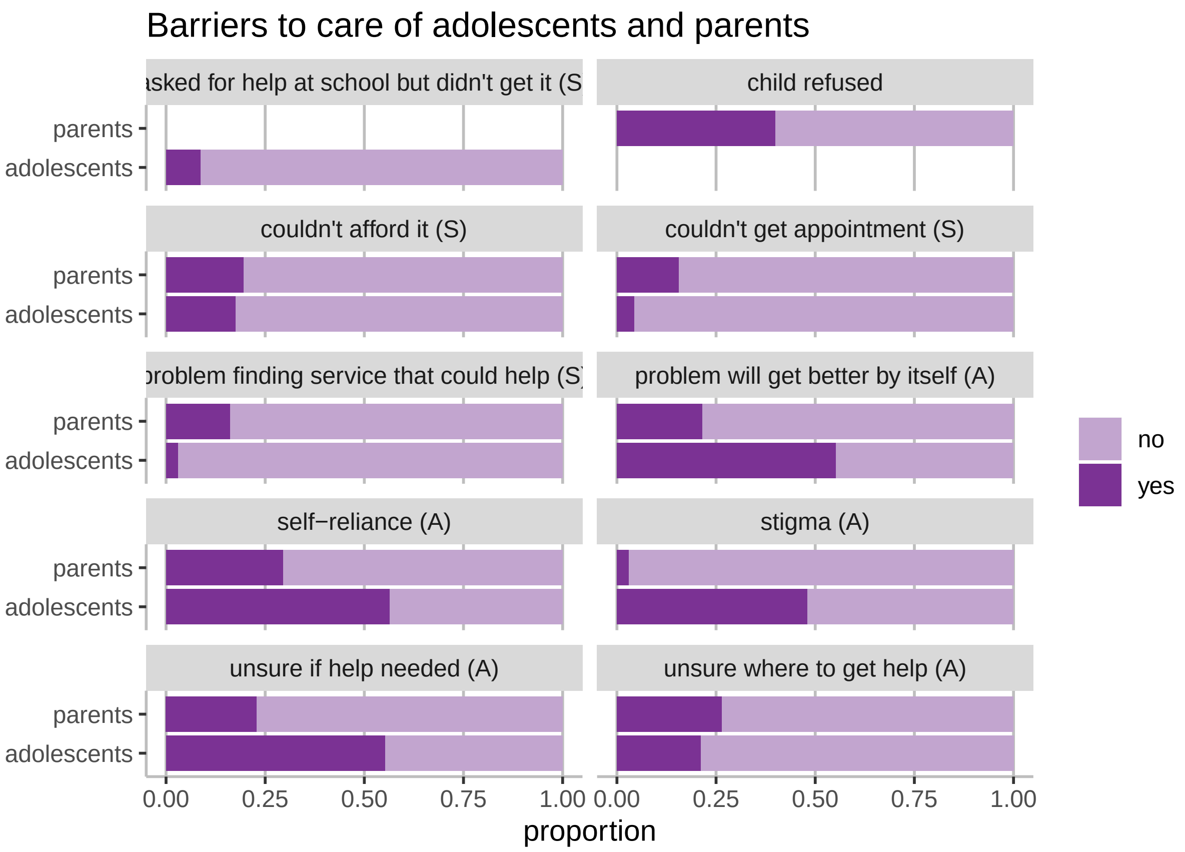


```
# without numbers before labels (01_, 02_, 03_) order not as i want it to be #### solution ####
YMM_barrier_long$question_f = factor(YMM_barrier_long$question,
                                     levels = c("stigma (A)", "self-reliance (A)",
                                                "unsure if help needed (A)","unsure where to get help (A
                                                "problem will get better by itself (A)",
                                                "problem finding service that could help (S)", "couldn't
                                                "couldn't get appointment (S)",
                                                "child refused", "asked for help at school but didn't ge

# plot with new order ####
ggplot(na.omit(YMM_barrier_long), mapping = aes(x= source, fill = noyes)) +
  geom_bar(position = "fill") +
  facet_wrap(~question_f, nrow = 5, ncol = 2) + coord_flip() + xlab("") + ylab("proportion") +
  ggtitle("Barriers to care of adolescents and parents") +
  scale_fill_manual("", values = c("#c2a5cf", "#7b3294")) +
  theme(panel.grid.major.x = element_line(colour = "grey"), panel.background = element_blank(),
        axis.line = element_line(colour = "grey"))
```

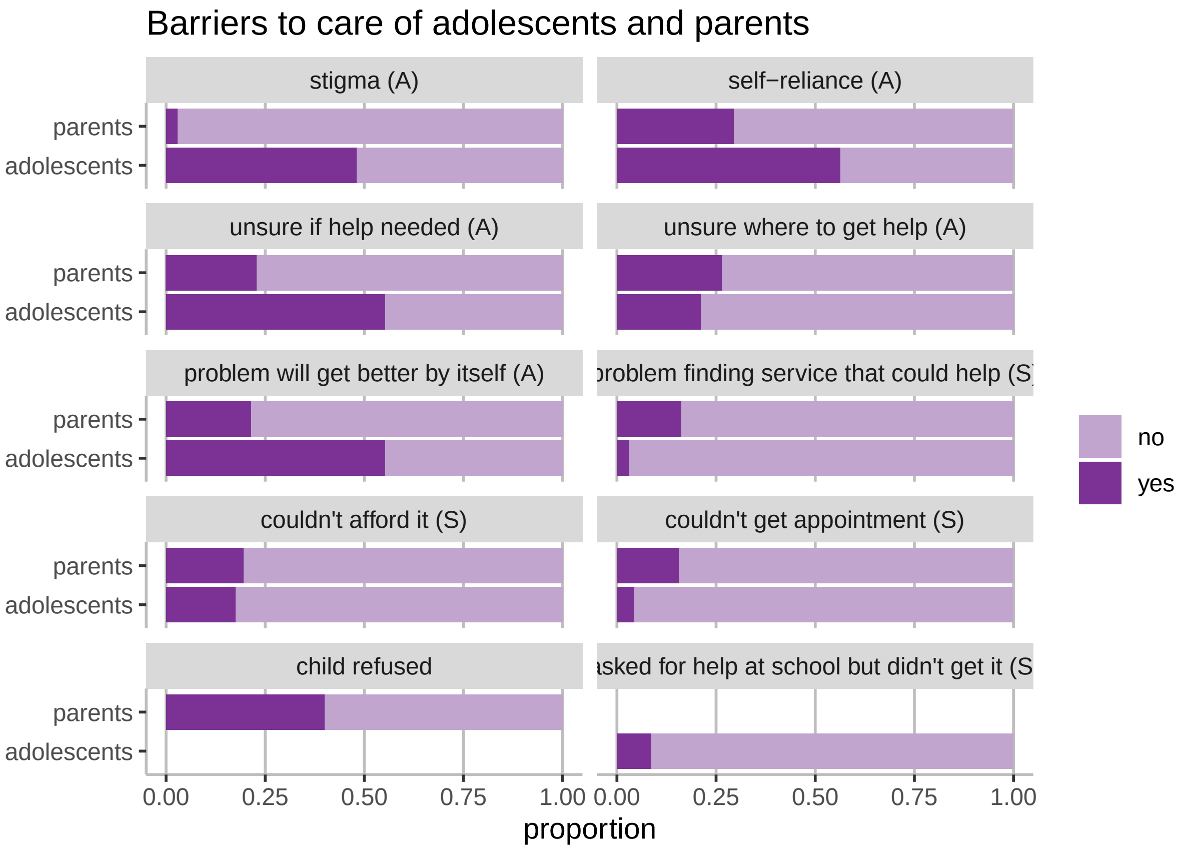


```
# plot with new order and grey bars for publication, no title, and percent instead of proportion ####
library(dplyr)
YMM_barrier_long_perc <- YMM_barrier_long %>%
  group_by(source,question_f,noyes) %>%
  summarise(count=n()) %>%
  mutate(perc=count/sum(count))

brks <- c(0, 0.25, 0.5, 0.75, 1)

ggplot(na.omit(YMM_barrier_long_perc), mapping = aes(x = source, y = perc, fill = noyes)) +
  geom_bar(position = "fill", stat = "identity") +
  scale_y_continuous(breaks = brks, labels = scales::percent(brks)) +
  facet_wrap(~question_f, nrow = 5, ncol = 2) + coord_flip() + xlab("") + ylab("percent") +
  scale_fill_manual("", values = c("#969696", "#252525")) +
  theme(panel.grid.major.x = element_line(colour = "grey"), panel.background = element_blank(),
        axis.line = element_line(colour = "grey"))
```

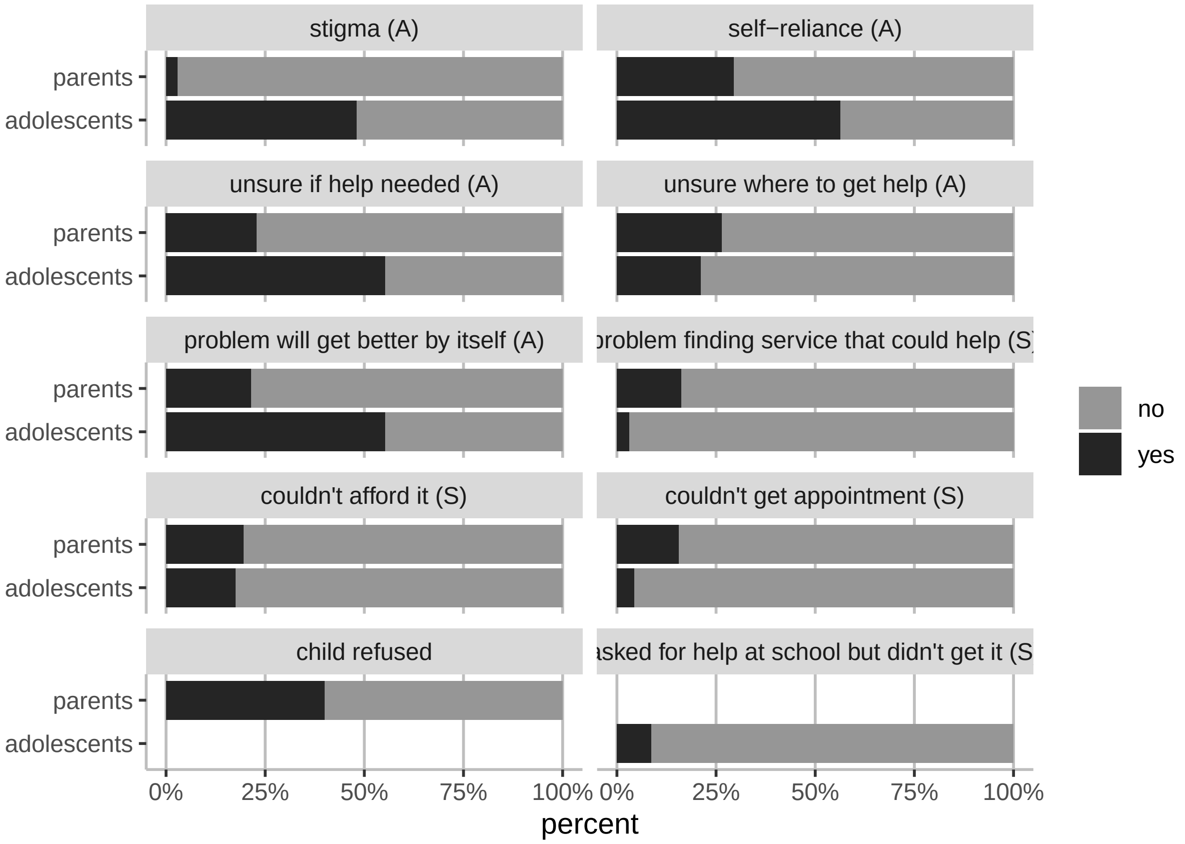


```
# rm data sets
rm(YMM_barrier_parents, YMM_barrier_adolescents, YMM_barrier)
rm(YMM_barrier_long_adolescents, YMM_barrier_long_parents)
```

Survey weighted

```
# chose variables for survey analysis
YMM_barrier_survey <- select(YMM, householdID, cluster, Age, YouthWeightC, par_selfrel:ado_school)

YMM_barrier_survey$par_school <- NA
YMM_barrier_survey$ado_refuse <- NA

# survey design
# one-stage cluster sample (we don't have variables for multistage sampling to include: https://www.sta

YMM_survey <- svydesign(id = ~cluster,
                        probs = NULL,
                        strat = NULL,
                        weight = ~YouthWeightC,
                        data = YMM_barrier_survey,
                        nest = FALSE)

# restrict survey design to subpopulation
YMM_surv_ado <- subset(YMM_survey, Age>=13)
```

```
# approach 2: estimates for ados and parents separately, then merge so we have two groups.
# parents
barriers_par <- svymean(~par_stigma + par_selfrel + par_unsneed +
```

```
                      par_unswhere + par_bettself + par_getserv +
                      par_cost + par_appoint + par_refuse,
                      YMM_surv_ado, na.rm = TRUE)

barriers_CI_par <- confint(svymean(~par_stigma + par_selfrel + par_unsneed +
                      par_unswhere + par_bettself + par_getserv +
                      par_cost + par_appoint + par_refuse,
                      YMM_surv_ado, na.rm = TRUE)) # 95% confidence intervalls instead of SE

barriers_par <- cbind(barriers_par, barriers_CI_par)

barriers_par <- as_tibble(barriers_par)

barriers_par <- barriers_par[c(2, 4, 6, 8, 10, 12, 14, 16, 18), ] # chose only "yes" answers

par_school <- c(0, 0, 0) # add row to parent df for adolescent barrier "asked at school but didn't get
barriers_par <- rbind(barriers_par, par_school)

barriers_par$question <- c("Stigma (A)", "Handle problem on my own (A)", "Unsure if help needed (A)",
                       "Unsure where to get help (A)", "Problem will get better by itself (A)",
                       "Problem finding service that could help (S)",
                       "Couldn't afford it (S)", "Couldn't get appointment (S)", "Child refused",
                       "Asked for help at school but didn't get it (S)")

barriers_par$group <- "Parent"

# rename couple of variables
barriers_par <- plyr::rename(barriers_par, c("barriers_par"="estimate", "2.5 %"="lower", "97.5 %"="uppe

# adolescents
barriers_ado <- svymean(~ado_stigma + ado_selfrel + ado_unsneed +
                        ado_unswhere + ado_bettself + ado_getserv +
                        ado_cost + ado_appoint + ado_school,
                        YMM_surv_ado, na.rm = TRUE)

barriers_CI_ado <- confint(svymean(~ado_stigma + ado_selfrel + ado_unsneed +
                        ado_unswhere + ado_bettself + ado_getserv +
                        ado_cost + ado_appoint + ado_school,
                        YMM_surv_ado, na.rm = TRUE))

barriers_ado <- cbind(barriers_ado, barriers_CI_ado)

barriers_ado <- as_tibble(barriers_ado)

barriers_ado <- barriers_ado[c(2, 4, 6, 8, 10, 12, 14, 16, 18), ]

ado_refuse <- c(0, 0, 0) # add row to adolescent df for parent barrier "child refused"
barriers_ado <- rbind(barriers_ado, ado_refuse)

barriers_ado$question <- c("Stigma (A)", "Handle problem on my own (A)", "Unsure if help needed (A)",
                       "Unsure where to get help (A)", "Problem will get better by itself (A)",
                       "Problem finding service that could help (S)",
                       "Couldn't afford it (S)", "Couldn't get appointment (S)",
```

```
                        "Asked for help at school but didn't get it (S)", "Child refused")

barriers_ado$question = factor(barriers_ado$question,
                                levels = c("Stigma (A)", "Handle problem on my own (A)", "Unsure if help
                                         "Unsure where to get help (A)", "Problem will get better by its
                                         "Problem finding service that could help (S)",
                                         "Couldn't afford it (S)", "Couldn't get appointment (S)", "Chil
                                         "Asked for help at school but didn't get it (S)")) # same order

barriers_ado$group <- "Adolescent"

# rename couple of variables
barriers_ado <- plyr::rename(barriers_ado, c("barriers_ado"="estimate", "2.5 %"="lower", "97.5 %"="uppe

# merge ado and parent data sets by question
barriers_both <- rbind(barriers_par, barriers_ado)

# order of factors messy again (doesn't inherit order specified above). Display will be from bottom to
barriers_both$question <- factor(barriers_both$question,
                                  levels = c("Asked for help at school but didn't get it (S)", "Child re
                                             "Couldn't afford it (S)", "Problem finding service that cou
                                             "Problem will get better by itself (A)", "Unsure where to g
                                             "Unsure if help needed (A)", "Handle problem on my own (A)"

# graph
brks <- c(0, 0.1, 0.2, 0.3, 0.4, 0.5, 0.6) # specified in original values
dodge <- position_dodge(width=0.9) # to make the error bars narrower

ggplot(barriers_both, mapping = aes(x = question, y = estimate, fill = group)) +
  geom_col(position = "dodge") +
  geom_errorbar(aes(x=question, ymin = lower, ymax = upper), colour = "grey",
              position = dodge, width = 0.25) +
  coord_flip() + xlab("") + ylab("") +
  scale_y_continuous(breaks = brks, labels = scales::percent(brks, accuracy = 1)) +
  scale_fill_manual("", values = c("#969696", "#252525")) +
  theme(panel.grid.major.x = element_line(colour = "grey"), panel.background = element_blank(),
        axis.line = element_line(colour = "grey"))
```

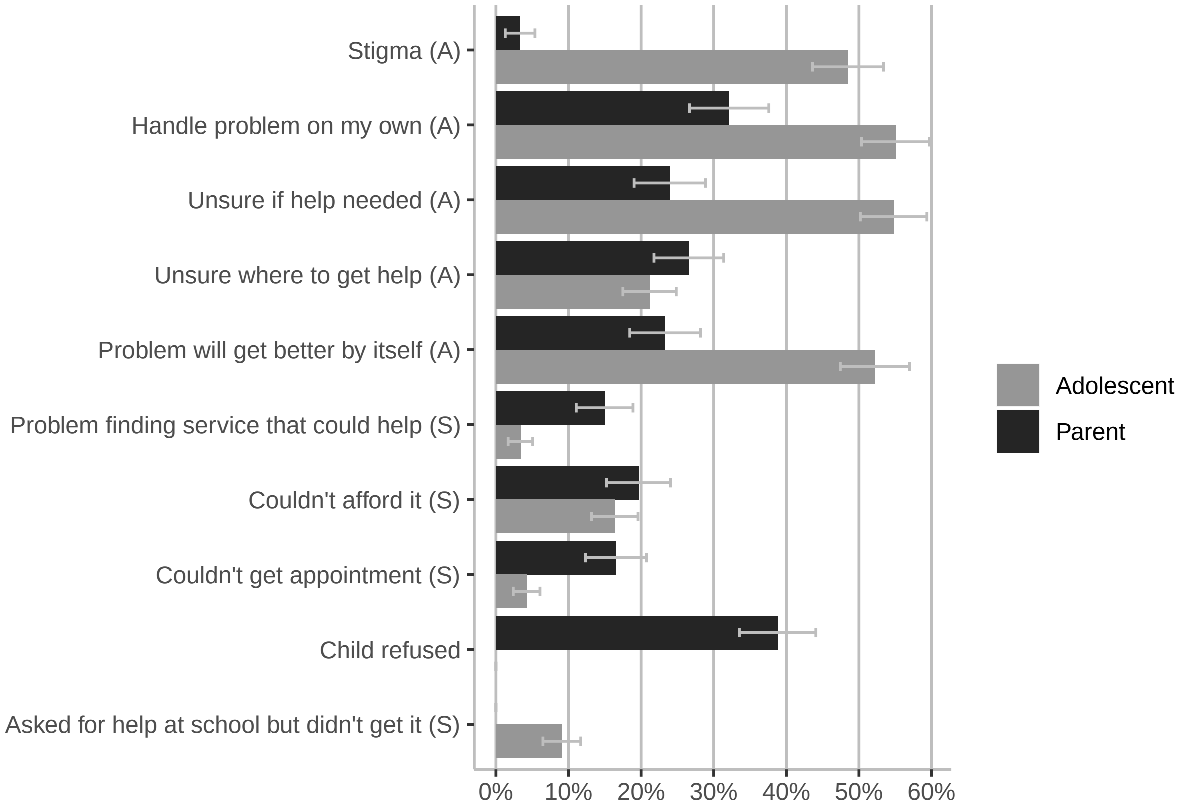
Stigma (A)
Handle problem on my own (A)
Unsure if help needed (A)
Unsure where to get help (A)
Problem will get better by itself (A)
Problem finding service that could help (S)
Couldn't afford it (S)
Couldn't get appointment (S)
Child refused
Asked for help at school but didn't get it (S)
0%
10%
20%
30%
40%
50%
60%
Adolescent
Parent
